# Feeling of hand deformation as a monkey's hand: an experiment on a visual body with discomfort and its algebraic analysis

**DOI:** 10.3389/fnins.2023.975597

**Published:** 2023-07-10

**Authors:** Yang Ruijia, Hirokazu Sakura, Yukio-Pegio Gunji

**Affiliations:** Department of Intermedia Art and Science, School of Fundamental Science and Technology, Waseda University, Tokyo, Japan

**Keywords:** sense of ownership, body image, body deformation, rough set, lattice

## Abstract

While there are many studies in which body ownership can be transferred to a virtual body, there are few experimental studies of how subjects feel about their own bodies being deformed since a real body cannot be deformed. Here, we propose such an experimental setup, in which a twisted hand is diagonally viewed from behind, which is called a “monkey's hand.” Although the subject cannot see the thumb hidden behind his or her arm, he or she feels that the monkey's hand has an ambiguous thumb that functionally never exists but structurally exists. This ambiguity is consistent with experimental results on proprioceptive drift, by which the deformation of the hand is measured. The ambiguity of the presence and absence of the thumb is finally analyzed with a specific algebraic structure called a lattice. This can help us understand disownership as being different from the absence of ownership.

## 1. Introduction

The body is considered to play a positive and active role in interfacing between consciousness and the world surrounding a subject. In that sense, we can utilize the body to explore the environment. However, the body can also show the negative and passive implications, such as when it is injured or paralyzed. Since such feelings are destined to be accepted even if they are not preferable, these feelings are passive and negative. In this study, we focus on negative and passive feelings in the body.

The demand for active and positive implications for the body is also found in the study of bodily sensations. An active and positive implication is defined as a feeling that the subject can use the body to explore the environment and that this body is consistent with the subject's own healthy body. Research on bodily sensations has been broadly divided into a sense of ownership (SoO) and sense of agency (SoA) (Gallagher, 2000; Botvinick, 2004; Haggard and Eltam, 2010; Tsakiris, 2010). SoO is the feeling that the body is certainly one's own, and SoA is the feeling that the cause of the movement of the body is certainly one's own. However, SoO is not considered to be a single basic sensation but a comprehensive judgment (similar to the taste of wine) for various afferent signals (Ehrsson, 2020). In these studies, the subject may feel that his or her hand moves freely and is no longer his or her own; this research began with conventional research on alien hands (Hassan and Josephs, 2016). The question of how bodily sensations are formed is based on the two speculations that having bodily sensations has active and positive implications and that the formation of bodily sensations requires the integration of multiple sensations. These ideas have emerged as important to the intelligent body as a system that deals with the real world. The positive and active implication of the body is consistent with a healthy body that a subject can move as his or her own body. In that sense, the positive and active implication of the body is directly related to the sense of agency. Whether the virtual body can be moved or not, the subject is a healthy person who can move his or her own part of the body and that implies that the sense of ownership is also related to the positive and active implication of the body. The seminal experiment on this question is the rubber hand illusion (Botvinick and Cohen, 1998).

A rubber left-hand model is placed in front of the subject, and the subject's left hand is placed to the left of the rubber hand, occluded by a screen. The subject is instructed to gaze at the rubber hand. In this situation, the rubber hand and the subject's hand, in the same position, are simultaneously rubbed by a paintbrush. After that, the subject answers a subjective questionnaire. The subject is asked to indicate the position of his or her left hand before and after the stimulation, and the proprioceptive drift is calculated as the difference between these measurements. While this drift is frequently considered to be correlated with the sense of ownership, it has also been reported that subjective ratings and drift are dissociated (Rohde et al., 2011). It was concluded that the sense of ownership of the hand results from the integration of visual and tactile sensations. This has been verified in accordance with the principle of unity in space (Stein and Stanford, 2008).

The results of the asynchronous condition were examined in more detail, and it was reported that the sense of ownership decreased significantly when the time lag between the visual and tactile sensations exceeded 300 ms (Shimada et al., 2014) and was almost lost when it reached 500–1,000 ms. Spatial deviations also have tolerances that give subjects a sense of ownership (Tsakiris and Haggard, 2005), and such deviations have been reported to exist both horizontally and vertically (Lloyd, 2007; Kalckert and Ehrsson, 2014a). In addition, the direction of the brush is the same (Gentile et al., 2013), the position of the rubber hand is in a position that is not unreasonable for rotation (Ide, 2013), and there are congruent orientations and identical types of tactile sensation (Ward et al., 2015). It has also been experimentally clarified that such factors are conditions for acquiring a sense of ownership. There is also an allowable range of sense of ownership related to the deformation of the hand itself.

It has been found from various experiments that the important point is not the material, such as wood or metal, but the outer shape (Kalckert and Ehrsson, 2014a,b). Hand deformation can be dealt with in various ways by using VR with a head-mounted display (HMD). Stretched arms in VR are known to result in feelings of ownership that exceed spatial tolerance, which is interpreted as the brain being plastic enough to cope with slow arm stretching (Kilteni et al., 2012). It has been experimentally found that it is possible to acquire a sense of ownership of an invisible hand (Guterstam et al., 2013). In this case, a movement that traces the contour of the outer shape of the hand is needed, and it should not deviate from the permissible range regarding the outer shape.

In addition, experiments with a rubber hand can be extended to the whole body; subjects can feel a sense of ownership of a mannequin and a virtual body in VR, and a sense of ownership can also be obtained for an entire body that is a transparent human (Slater et al., 2010; Preston et al., 2015). It is thought that the sense of ownership is acquired not only through the integration of the visual sense and tactile sense but also through the integration of the tactile sense and proprioceptive sense or the integration of the visual sense and kinesthetic sense (Ehrsson, 2020).

Electrophysiological studies in macaque monkeys reveal the presence of single neurons in the prefrontal cortex and parietal cortex, especially in the cortex that lines the premotor cortex and intracranial groove, in response to all visual, tactile, and proprioceptive sensations (Graziano et al., 2004). Macaque monkeys are considered candidates for the integration of multisensory stimuli. In humans, studies using fMRI have found regions in the frontal and parietal lobes that respond to both visual and tactile stimuli. The hypothesis that the acquisition of physical ownership is due to the integration of multisensory stimuli is further supported (Makin et al., 2007; Gentile et al., 2013).

The above findings regarding the sense of ownership of the hand reveal the following. First, there is an acceptable range in the conditions for acquiring a sense of ownership. Second, when all or multiple parts, such as tactile, visual, proprioceptive, and kinesthetic sensations, are integrated, it is easy to obtain a sense of ownership even for things that are not an actual physical body. Third, even for deformations of objects such as hands realized in VR, humans will become accustomed to these deformations as long as sufficient time is taken for the deformations. The above three points indicate that the sense of ownership is flexible, and physical sensations can be transferred to other things, although there are restrictions.

The notion of an embodied mind (Clark, 1998; Varela et al., 2017) reveals that the body acts as an interface between logical intelligence and the real world since the body can realize what is called morphological computing (Pfeifer and Bongard, 2007). When the rubber skin of a robot's hand can contribute to grasping an egg without breaking the egg, one can say that the body as the skin morphologically, rather than logically, computes the degree of power needed to grasp the egg, that is, morphological computing plays an essential role in embodied intelligence.

The neurocognitive model of body ownership (Tsakiris, 2010; Ehrsson, 2020) is consistent with the notion of the embodied mind. They are both based on optimization in a world consisting of repeated experiences (i.e., a stationary world). Discomfort deviating from the experienced world is excluded from that framework. In the sense of optimization, constructed body ownership has active and positive implications. On the other hand, discomfort with body image must sometimes be accepted for one's own body, that is, there are negative and passive implications of body ownership. Discomfort does not imply a lack of body ownership. While body ownership is reduced for incongruent rubber hands (Ehrsson, 2020), that situation never reveals discomfort. In contrast, if the subject's own real hand is incongruent, there is discomfort and disownership notwithstanding the presence of the sense of agency (Nishiyama et al., 2015) that is not a lack of body ownership (de Vignemont, 2011).

People with autism spectrum disorder (ASD) often consider themselves abstract. They have a weak sense of the body. A hug machine that tightens the body on the left and right is known to be effective in giving a sense of security to people with ASD, but this may be because the physical inconvenience of an immobile body makes it possible to feel the existence of the body for the first time (Minoura et al., 2019, 2020b).

Discomfort contributes to both body ownership and disownership. Since there is discomfort if a subject's own hand is movable but incongruent, this can lead to disownership accompanied by a sense of agency (Nishiyama et al., 2015). If the rubber hand is replaced by the experimenter's real hand in the rubber hand illusion paradigm, a subject can experience a movable but uncontrollable hand with discomfort, which leads to body ownership with discomfort (Minoura et al., 2020a). These experiments are set up by using a real hand. In these studies, subjects feel disownership, in the sense that the body ownership carried by the subject's own body is totally different from body ownership in everyday life and/or that body ownership is maintained notwithstanding real hand discomfort. In addition, there are reports of paralyzed bodies and bodies with disabilities that clearly indicate the existence of the body due to their discomfort (Giummarra et al., 2012). This is also true for the real body.

In contrast, body discontinuity in VR space leads to the situation that a subject does not experience body ownership despite experiencing a sense of agency (Tieri et al., 2015). A lack of experience of ownership does not imply the experience of loss of ownership. In this sense, this is not disownership. The extreme case of body ownership accompanied by discomfort is the phantom limb case (Nikolajsen and Christensen, 2015). Synchronous touching of the ear and a paralyzed arm can lead to much more body ownership in paralyzed bodies (Pazzaglia et al., 2019). This implies the recovery of body ownership accompanied by discomfort. This case also involves the real body. It is very difficult to detect discomfort that is not in a real body but in a virtual body.

Our key idea is the significance of discomfort (i.e., passive and negative implications). In fake bodies such as rubber hands or VR hands, body ownership never involves discomfort. If a subject feels discomfort, he or she cannot acquire body ownership of a fake body. Otherwise, he or she can acquire body ownership. In that scheme, discomfort and body ownership never coexist. In contrast, a subject's own hand that is movable but incongruent (Nishiyama et al., 2015), real hand illusions (Minoura et al., 2020a), and body ownership of paralyzed bodies (Pazzaglia et al., 2019) involve discomfort, whether body ownership is acquired or not. Strictly speaking, a subject feels not loss of ownership but disownership in his or her own hand if it is movable but incongruent (Nishiyama et al., 2015). Since disownership is neither ownership nor loss of ownership, it is a feeling of a body with a kind of discomfort. Because that feeling is different from ordinary body ownership, a subject frequently claims that he or she does not feel body ownership. Disownership generated in a real body is different from the loss of ownership; rather, it reflects ambiguity regarding the dualism of ownership and loss of ownership. In this study, we propose an approach to the sense of body ownership that is based on passive and negative implications, using a case in which the feeling that a part of the body is missing and deformed can be instantly induced. This will be discussed in the next section.

To evaluate body ownership with discomfort and/or ambiguity regarding the absence or presence of the thumb, we introduce a lattice derived from rough set theory that enables data analysis based on discernibility, which is taken as an equivalence relation (Pawlak, 1981, 1982; Polkowski, 2002). In rough set theory, a regularity called a decision rule is obtained for data in the form of a decision table, and the relative reduct is one of the most important regularities. Although a relative reduct cannot be obtained (Skowron and Rauszer, 1992), many algorithms have been proposed to obtain candidates for the relative reduct (Hu et al., 2000, 2008; Tan et al., 2005; Xu et al., 2007). In addition, there are many generalizations of rough sets in data analysis (Ziarko, 1993; Yao and Lin, 1996; Greco et al., 2001; Zhu, 2007). Recently, rough sets have been developed in the field of three-way decision-making (Yao, 2010, 2012, 2018).

Rough sets are applied not only to data mining but also to logic. In particular, the relationship between modal logic and rough set theory has been researched (Orlowska, 1984; Vakarelov, 1989; Järvinen, 2007a,b), and the relationship between a rough set and a lattice has been studied with a fixed point of the composition of lower and upper approximations (Yao, 2004a,b; Yao and Chen, 2006; Li et al., 2017). Independently of these studies, one of the authors found a lattice construction based on rough sets and proposed a representation theorem (Gunji and Haruna, 2010). In this study, we discuss various techniques of lattice theory (Davey and Priestley, 2002); in the preliminary section, we discuss lattices along with easily accessible citations.

## 2. Research objective: passive attitude

**Figure 1:**
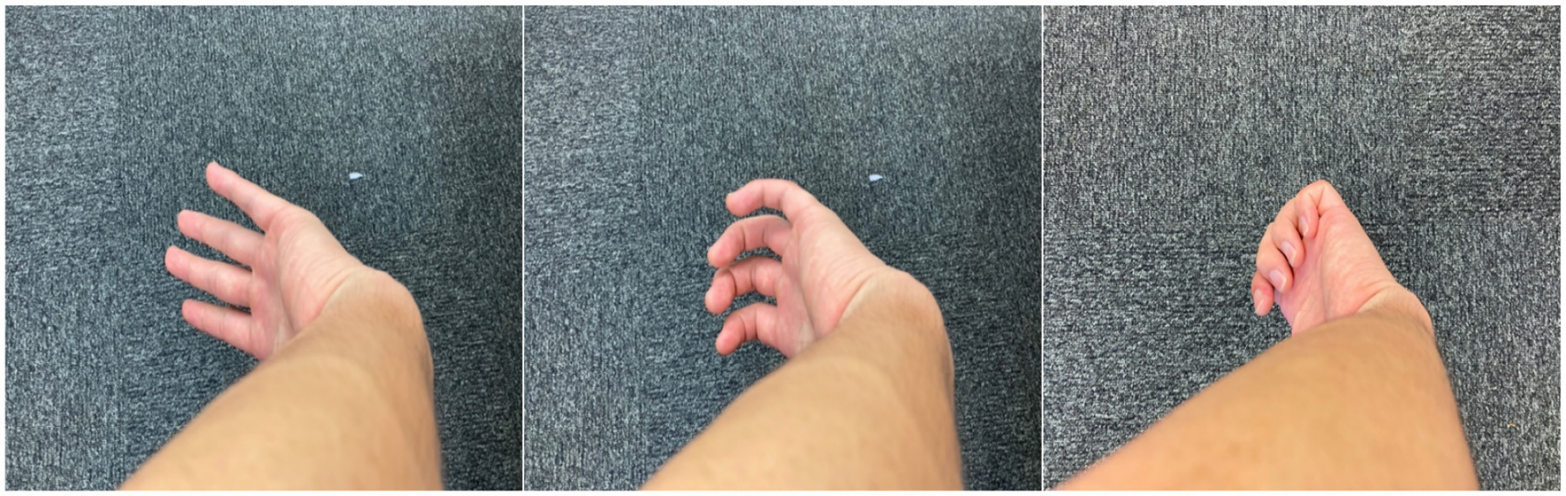
Various postures of the “monkey's hand.”

Here, we conducted an experimental study on the deformation of the hand as a pure illusion, called a “monkey's hand” in this study, without using HMD. This illusion can be achieved by simply turning the inside of the palm toward the outside of the body, extending the arm, and hiding the thumb in the shadow of one's arm while looking at the moving hand. The subject should judge whether the thumb is only hidden and is visually determined to have disappeared or deformed (Figure 1).

The monkey's hand illusion was found by one of the authors ~10 years ago, and Gunji (2013), which was published in Japanese, referred to it without experimental evaluation. Thus, the experimental study of the monkey hand illusion is first described in this study. In addition, we explain why we call this illusion the “monkey's hand illusion.” Compared to those of humans, the thumbs of apes and monkeys are far from the other four fingers, which are adapted to grasp trees. Only humans have a thumb close to the other four fingers, which constitute an organ dedicated entirely to manipulation (Marzke, 1992). Although the thumbs of apes and monkeys have a structural similarity with those of humans since the thumb is one of five fingers, there is little functional similarity. From the human point of view, monkeys and apes have a thumb with respect to structure but not with respect to function. This ambiguous status of the thumb is intrinsic to the monkey's hand illusion. We feel as if the palm were extended and deformed like that of a monkey in the monkey's hand illusion, and we feel as if the thumb were (functionally) lost and (structurally) exists. That is why we call this illusion the monkey's hand illusion.

The illusion of a monkey's hand is that it is one's own hand, so it is possible to move the finger freely, and the sensation of moving the finger and the visual sense are completely synchronized.

As a result, it feels as if the thumb were lost as an element of the hand but as if the thumb exists somewhere far from the palm. Although such a feeling is somewhat confusing, the illusion seems to reflect that the hand is similar to a monkey's hand, where the monkey's thumb is both present (since it is structurally similar to the human thumb) and absent (since it is not functionally similar to the human thumb; Figure 1). This illusion of the body does not indicate the freedom to transfer the sense of possession to an unknown deformed object but conversely has the negative implication that the thumb disappears and the palm deforms slightly.

Although it is difficult to accept that the subject's own hand is deformed to the monkey's hand, the deformation can be accepted along with strange and negative feelings. However, the effect of the illusion depends on the subject.

How will this finding change by creating an experimental system of approaches that give passive and negative implications to the sense of ownership? The objective of this study was to propose such a model experiment and determine the direction of the study. Since the palm illusion proposed here relies on vision, the way vision affects proprioceptive sensations is evaluated. In addition, since the active/passive approach “creates” a place/object to which the sense of ownership shifts, the possessed body or a part thereof is always treated as a dualistic evaluation of “existence/non-existence.” In contrast, in this experiment, the defect of the thumb is perceived in comparison with the five-fingered hand, and the reality of the deformed hand is felt. It should be possible to handle not only the dualistic evaluation of “existence/non-existence” but also absence, such as the judgment that “it should exist but not here.” Thus, whether the absence of the thumb is perceived is evaluated by a subjective questionnaire, and how it affects the illusion of the palm is discussed through a mathematical structure (lattice) (Davey and Priestley, 2002; Yao, 2004a; Gunji and Haruna, 2010).

## 3. Materials and methods

### 3.1. Participants

This experiment was conducted at Waseda University Nishi-Waseda Campus from June 11 to July 8 in 2021. The subjects were 32 healthy men and women aged 18–25 years, including 18 men and 14 women. The average age was 21.1 years. The subjects were recruited with a reward; the experiment was conducted after giving an overview in advance, and consent was obtained. The data for all 32 subjects were used as valid data for the analysis. We only experimented with the right hands (32 participants) regardless of the participant's dominant hand. We did not find any difference between the groups.

### 3.2. Experimental apparatus

**Figure 2:**
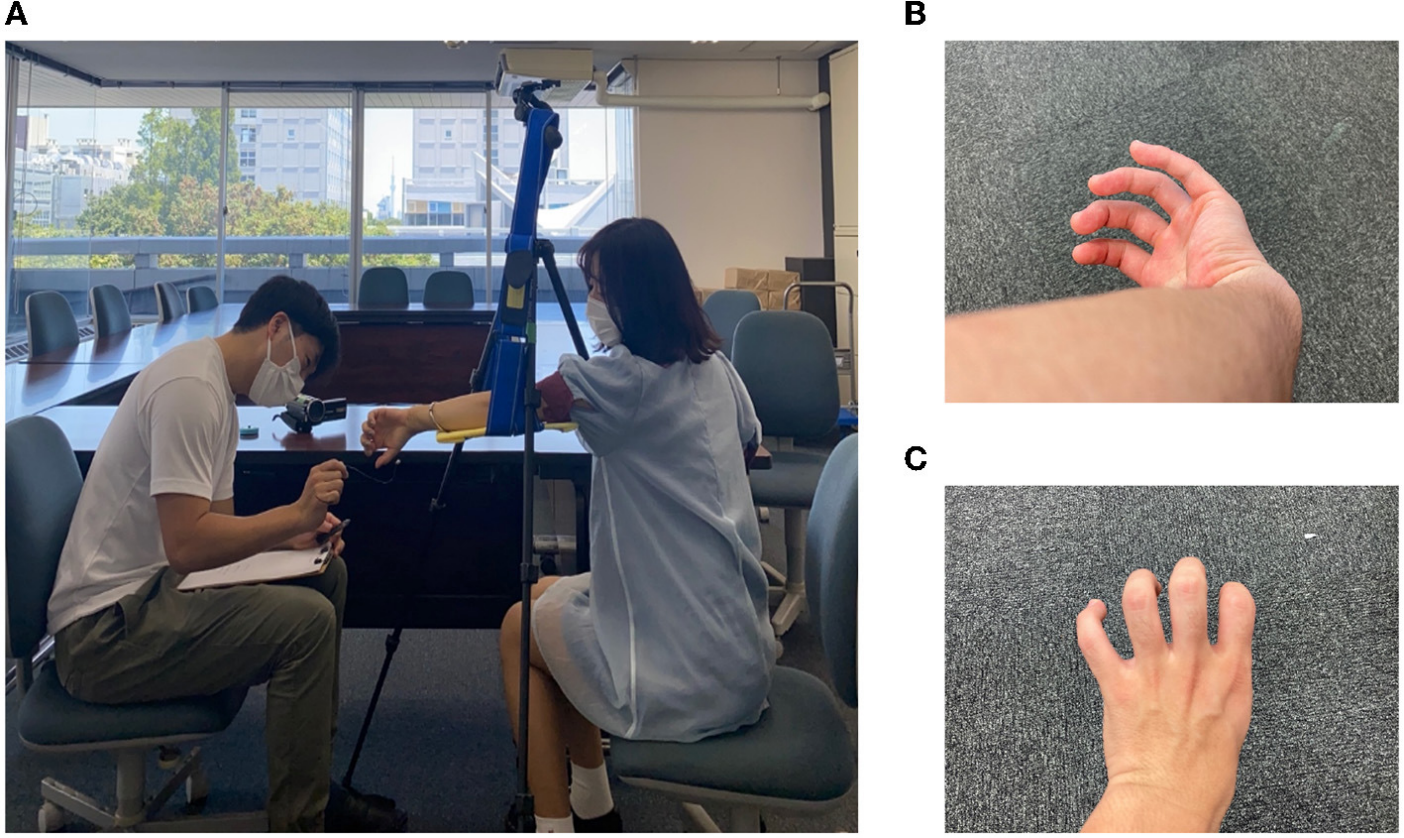
**(A)** Experimental setup. **(B)** Monkey's hand condition. **(C)** Hidden thumb hand condition.

The experimental equipment was set up as shown in Figure 2A. The equipment used in the experiment was as follows: a tripod, a plastic board, two cloths, a stopwatch (iPhone7plus), a video camera (Everio GZ-MG740, Victor), a cotton stick, a wire, a ruler, and a blue sticker. In the experiment, the experimenter sat on the left and the subject sat on the right, and the experiment was photographed from the front of the subject with a video camera.

### 3.3. The “monkey's hand” illusion

Here, we explain the illusion of the “monkey's hand” used in this experiment. If one twists one's left arm as shown in Figure 2B and takes a position where the thumb is hidden behind the arm and the four fingers and palm are visible, it is as if the thumb were lost. This can create the illusion that one has only four fingers. Since the hand looks like a monkey's or chimpanzee's hand (although monkeys and chimpanzees have five fingers), this hand state is named the “monkey's hand.” In each experiment, the subject creates this “monkey's hand” state and performs tasks.

Although there are individual differences, many of the subjects can easily feel that “the palm has become four fingers” rather than “the thumb is hidden and invisible,” as described below. Through the “monkey's hand,” the absence of the thumb and the deformation of the palm can be felt while ensuring a sense of ownership. Additionally, to clarify the meaning of the absence of the thumb, the “hidden thumb hand” was used as a control experiment for the “monkey's hand.” The “hidden thumb hand” was set as shown in Figure 2C. This is the state of the hand seen from the back side of the hand with the thumb folded inward. The “hidden thumb hand” is similar to the monkey's hand in that only the thumb is hidden, but it seems difficult to perceive the deformation of the hand here. By comparing the “monkey's hand” with the “hidden thumb hand,” we experimented with differences in subjective images and the structural significance of the absence of the thumb, and we quantified the results. In addition, by comparing the “monkey's hand with open eyes” with “that with closed eyes,” the drift of proprioceptive sensation was measured.

### 3.4. Experimental procedure

In the whole experiment, the subjects were divided into Groups A and B, and the order of the experiments was changed between Groups A and B, where the content of the experiments and tasks were the same. The way we separated the participants into Groups A and B was completely random. All the conversations were in Japanese. The time required for all the experiments was ~40 min. The flow of the experiments is shown in Table 1, which describes the experimental procedure. For instance, in Group A, first, the subject was asked to answer the preexperimental questionnaire. Second, the subject was asked to put his or her hand in the monkey's hand position and move his or her own fingers freely and then was asked to answer the subjective questionnaire. This is the main experiment (condition). Third, the subject was asked to make the folding thumb position and move his or her own fingers freely and then was asked to answer the same subjective questionnaire as used in the main experiment. This is the control experiment (condition). Fourth, the subject was asked to perform Experiment 1, which was conducted to measure the illusion of palm extension. Experiment 1 was performed in the monkey's hand position with open eyes (main condition) and with closed eyes (control condition). After a series of performances, the subject was asked to answer the postexperiment questionnaire for the normal monkey hand position. Fifth, the subject was asked to perform Experiment 2, which was conducted to measure the illusion of the hidden thumb extension. Experiment 2 was also performed in the monkey's hand position with open eyes (main condition) and with closed eyes (control condition). After a series of performances, the subject was asked to answer the postexperiment questionnaire for the reversed monkey hand position, which was different from the questionnaire used in Experiment 1. The difference between Groups A and B was simply the order of the experiments under the main condition and the control condition.

**Table 1:** Experimental flow.

**Group A**	**Group B**
Preexperiment questionnaire	Preexperiment questionnaire
↓	↓
Subjective task (main)	Subjective task (control)
↓	↓
Subjective questionnaire A	Subjective questionnaire B
↓	↓
Subjective task (control)	Subjective task (main)
↓	↓
Subjective questionnaire A	Subjective questionnaire B
↓	↓
Experiment 1 (a → b)	Experiment 1 (b → a)
↓	↓
Postexperiment questionnaire A (main)	Postexperiment questionnaire B (main)
↓	↓
Experiment 2 (a → b)	Experiment 2 (b → a)
↓	↓
Postexperiment questionnaire A (control)	Postexperiment questionnaire B (control)

Blanks in purple: experimental task; blanks in blue: questionnaire. The symbol a → b (b → a, respectively) represents the order of the task from a to b (from b to a, respectively) (a: monkey's hand position with open eyes; b: monkey's hand position with closed eyes).

#### 3.4.1. Preexperimental questionnaire

First, the subjects were asked to answer the preexperimental questionnaire as shown in Table 2. This questionnaire investigated the subject's attributes, such as gender, age, dominant hand, and sports history, to determine whether they were related to the experimental results. The same questionnaires were used for Group A and Group B.

**Table 2:** Contents of the “preexperiment questionnaire.”

**Age**	**__ years old**
Gender	
Dominant hand	Right·Left
Longest participation in a sport	Preschool/elementary school/junior high school/high school/university/____
	Period: ___ year
	item:
Most recent sport	Period: ___ ___~___ ___ _____Years
	item:
Frequency of regular exercise	Never·less than once a week·2–4 times a week·more than 5 times a week

#### 3.4.2. Experimental subjective questionnaire

**Figure 3:**
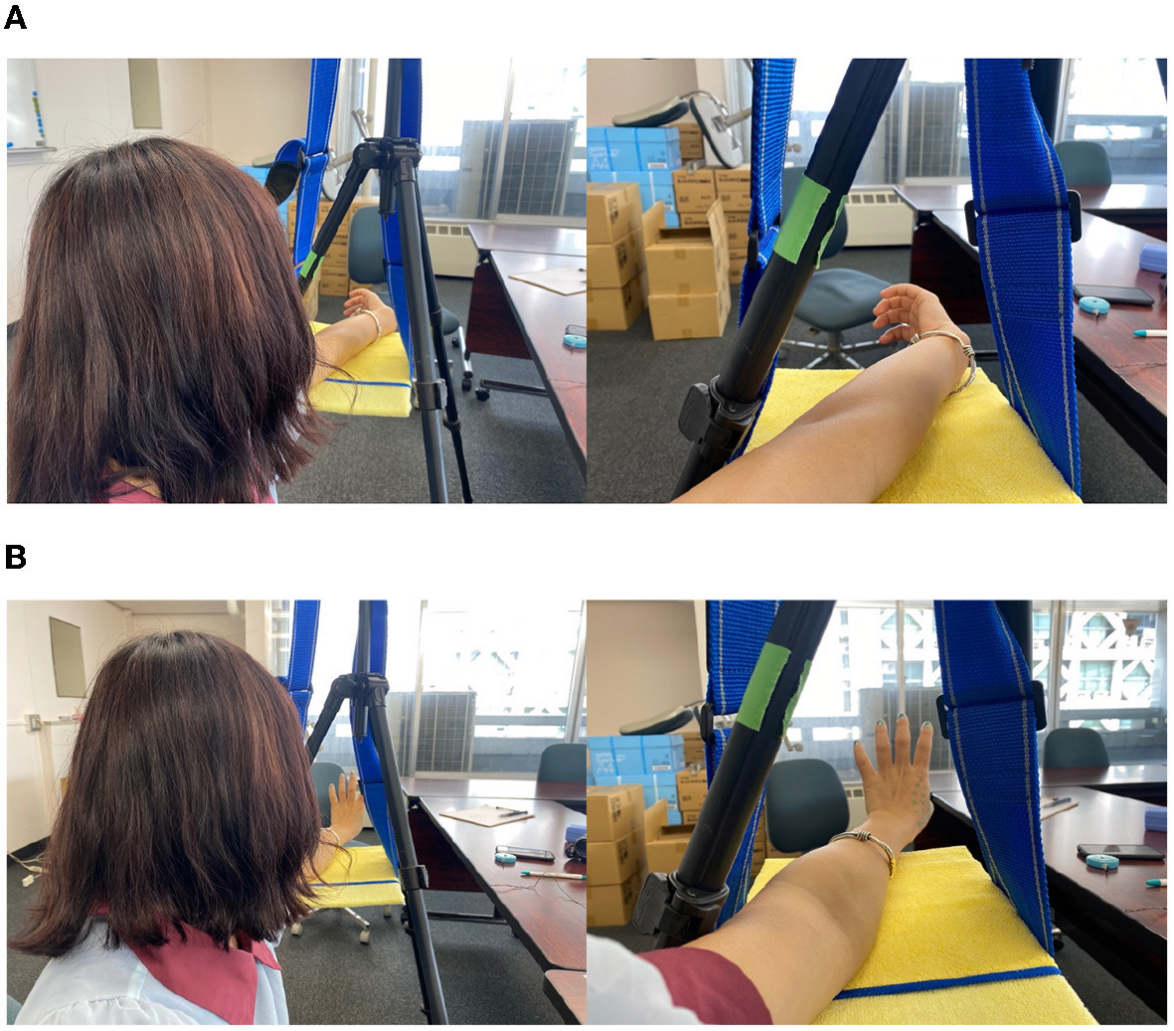
Subjective task **(A)** in the main experiment and **(B)** in the control experiment.

After answering the preexperiment questionnaire, the subjects sat in a predetermined position and performed each experiment. First, the subjects were asked to perform a subjective task. In Group A, the left arm was placed on a hanging board, as shown in Figure 3A, for the main experiment to create a “monkey's hand” state. Then, the four fingers were moved freely for 20 s while looking at the hand. We aimed to establish the illusion through visual short-term memory in these 20 s (Atkinson and Shiffrin, 1968). After that, the subjects were asked to answer “Subjective Questionnaire A,” as shown in Table 3.

**Table 3:** Contents of “subjective questionnaire A.”

**Q1**	**Because it looks like a four-fingered hand, I realize that I don't have a thumb**
Q2	I feel that my thumb doesn't exist
Q3	Being able to see the middle finger has nothing to do with being able to see the other fingers
Q4	I feel that the index finger does not exist
Q5	Seeing the ring finger is sufficient to notice the existence of the ring finger
Q6	Fingers other than the visible index finger are independent of seeing the index finger
Q7	The existence of the little finger is noticed only by seeing the little finger
Q8	I can see my thumb

Next, the subjects performed a control experiment, as shown in Figure 3B. With the back of the hand and four fingers visible in the “hidden thumb hand” state, the four fingers were moved freely for 20 seconds in the same way, and “subjective questionnaire A,” as shown in Table 3, was answered again. Group B performed the subjective tasks of the control experiment before those of the main experiment. This is the difference between Group A and Group B. The order of the questions in “Subjective Questionnaire B” was changed from that in “Subjective Questionnaire A,” but the questions themselves were the same.

Regarding Subjective Questionnaire A, Q1 is intended to examine the degree of the illusion, such as whether one's hand feels like four fingers in the state of a monkey's hand, and Q2 is intended to determine whether it feels as if the thumb is gone. Additionally, Q3, Q5, Q6, and Q7 are intended to confirm whether each finger other than the thumb can be recognized as that finger independently of the other fingers. Q4 and Q8 are dummy questions used to detect an answer that does not meet the research aims. We designed the questions to test the feasibility of the experiment.

#### 3.4.3. Experiment 1

**Figure 4:**
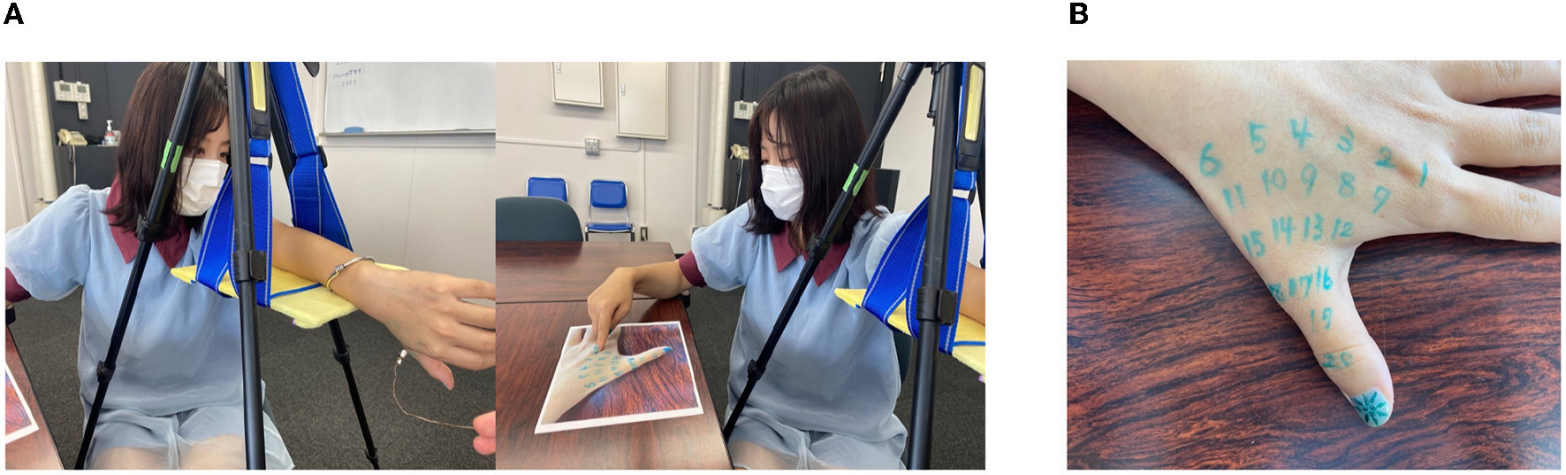
**(A)** Experiment 1 (main experiment). **(B)** Numbers written on the back of the hand.

After answering the “subjective questionnaire,” Experiment 1 was performed. In this experiment, while the subject looked at the palm and four fingers in the state of the monkey's hand, as shown in Figure 4A, the experimenter touched a number written on the subject's hand with a cotton swab. Then, the subject was asked to guess what number was touched and gave the answer (Figure 4B). The number was selected at random, and it depended on the size and shape of the subject's hand. The subject responded by looking at a preprinted photograph of his or her hand with numbers written on it and comparing it with the tactile sensation when touched.

In the control experiment, the same operation as that in the monkey's hand position was performed with the subject closing his or her eyes, and the setup of the experiment was the same as that shown in Figure 4A. The aim was to estimate how the degree of illusion in terms of deformation of the palm is achieved visually. The number touched was randomly determined for each subject in advance.

In Experiment 1, the deviation between the actual touched position and the position where the subject felt touched was measured. This deviation indicates the proprioceptive drift regarding the body position, especially the deformation of the palm. The main experiment and the control experiment were performed alternately 10 times each, for a total of three sets (60 times in total). However, Group A started with the main experiment, and Group B started with the control experiment. Additionally, the four fingers were moved for 7 s between sets to recreate the illusion state to clear the illusion and reset the device (this operation was similar to the subjective task under the main experiment). After Experiment 1 was completed, the subject was asked to answer Postexperiment Questionnaire A (Table 4) for the normal monkey eyes position. In Group B, after Experiment 1 was completed, the subject was asked to answer Postexperiment Questionnaire B (Table 5) for the reversed monkey hand position mentioned in the next section.

**Table 4:** Contents of “postexperiment questionnaire A (main).”

**Q1**	**After moving my four fingers freely, I felt like my thumb wasn't there**
Q2	I felt that the hand was originally a four-fingered palm, rather than a five-fingered hand with the thumb missing
Q3	I felt that the four-fingered palm was my palm
Q4	I felt that my four fingers were free to move
Q5	I felt that the arm with the palm and four fingers was newly added
Q6	I felt that my invisible thumb had moved somewhere else on my body
Q7	I felt that the skin of my four fingers had a different texture than my palms
Q8	I felt that my palm was covered with a four-fingered palm
Q9	In Experiment 1, I felt that I was touching something other than my own hands
Q10	I felt pain when I touched my hand in Experiment 1

**Table 5:** Questionnaire content of “postexperiment questionnaire A (control).”

**Q1**	**After moving my four fingers freely, I felt like my thumb wasn't there**
Q2	I felt that the hand was originally a four-fingered palm, rather than a five-fingered hand with the thumb missing
Q3	I felt the four-fingered palm was my palm
Q4	I felt that my four fingers were free to move
Q5	I felt that the arm with the palm and four fingers was newly added
Q6	I felt that my invisible thumb had moved somewhere else on my body
Q7	I felt that the skin of my four fingers had a different texture than my palms
Q8	I felt that my palm was covered with a four-fingered palm

#### 3.4.4. Experiment 2

**Figure 5:**
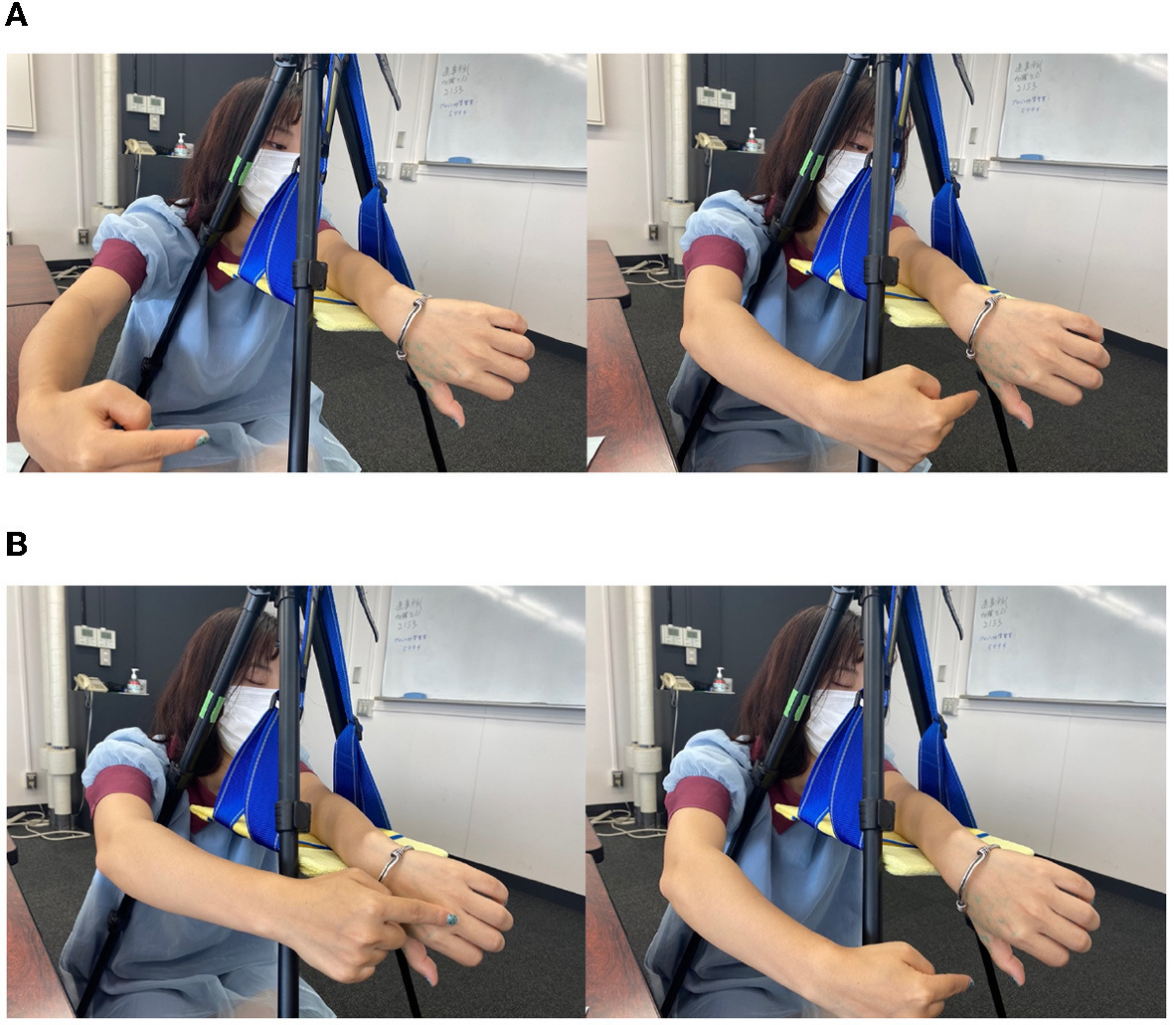
**(A)** Experiment 2 (main experiment). **(B)** Experiment 2 (main experiment).

Subsequently, Experiment 2 was performed. In this experiment, the subject touched the tip of the thumb of the left hand with the index finger of the right hand while looking at the palm and four fingers in the state of the “monkey's hand” (Figure 5A). Before starting Experiment 2, we placed a small blue dot sticker on both the tip of the index finger of the right hand and the tip of the thumb of the left hand. During Experiment 2, the subject tried to use the right-hand dot sticker to touch the left-hand dot sticker. We measured the distance between the two dots.

The subject stopped his or her hand by saying “yes” at the moment when he or she thought they were touching, and the experimenter measured the distance between the tip of the index finger of the right hand and the tip of the thumb of the left hand. In the control experiment, the subject performed the same operation with eyes closed to determine the sense of body ownership (SoO) without the visual sense. However, in the control experiment, the starting position was with the index finger of the right hand placed on the third joint of the middle finger of the left hand (Figure 5B). The main experiment and the control experiment were performed alternately, three times each (six times in total). However, Group A started with the main experiment, and Group B started with the control experiment.

**Figure 6:**
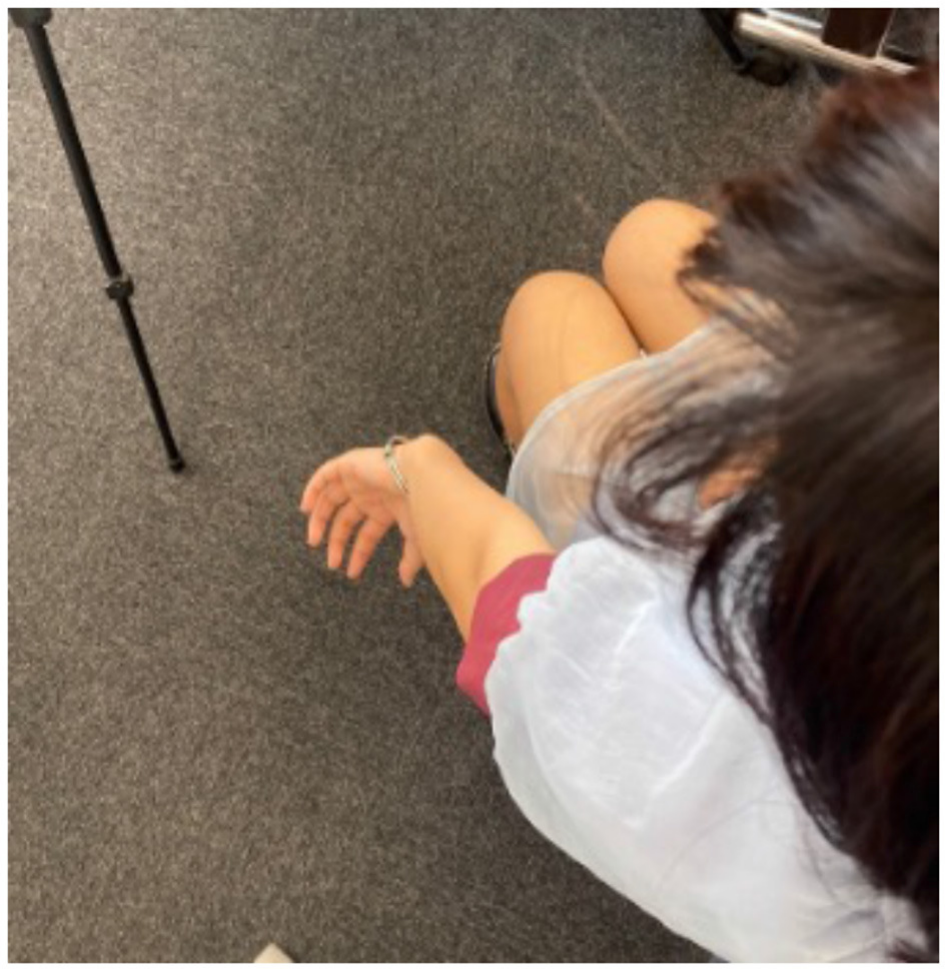
Posture when responding to the postexperiment questionnaire (control).

After Experiment 2 was over, in Group A, the subjects answered Postexperiment Questionnaire B (Table 5). The subjects responded by taking a position in which all five fingers and the palm could be seen, as shown in Figure 6 below. That position is called the reversed monkey hand position. In Group B, after Experiment 2 was completed, the subject was asked to answer Postexperiment Questionnaire A (Table 4) for the normal monkey hand position.

### 3.5. Lattice analysis

Although the monkey's hand illusion in this manuscript is presented in the literature for the first time, our analysis is strongly connected with the property of the illusion itself. The illusion is not so simple that it can be verified whether the modified hand is the subject's own or not. While the illusion involves contradictory properties, the illusion is genuine. It feels as if the thumb exists and does not exist. An illusion with contradictory properties is normally regarded as a non-well-defined illusion that cannot be verified. However, properties that are contradictory in normal classical logic do not imply a contradiction in alternative logic. Therefore, we analyzed the logical structure of the illusion indicated in our manuscript and showed that contradictory properties can be allowed in that logic. One reviewer who stated that he or she was a mathematician said that the application of lattice theory and algebra was adequate. In fact, showing a strong connection between phenomena and analytical methods can play an essential role in neuroscience.

In this study, we analyze the logical structure of the hand by using a subjective questionnaire. Therefore, we first explain the lattice and then describe how to use it for analysis.

Before providing a detailed definition, we start with a brief short history of lattice theory. Algebraic logic was proposed by George Boole (Boole, 1847, 1854) and was completed by Birkhoff's lattice theory (Birkoff, 1940). A lattice is an ordered set that is closed with respect to specific binary operations, join and meet (Davey and Priestley, 2002). Although elements in a set have no structure, elements in an ordered set have a specific structure called an order. While there are various sets equipped with structures called groups, rings, and fields, lattices are the simplest structured set (Passman, 2011). A lattice called an orthomodular lattice was developed not only for Boolean algebra but also for quantum logic (Birkhoff and von Neumann, 1936; Greechie, 1971; Maeda, 1980; Kalmbach, 1981, 1983; Khrennikov, 2001). Since quantum mechanics is used as information science to explain decision-making and cognitive illusions (Khrennikov, 2001, 2010; Aerts, 2009; Busemeyer and Bruza, 2012; Aerts et al., 2019; Ishwarya and Cherukuri, 2020), the way quantum logic is derived in cognition has recently been studied (Gunji and Haruna, 2022; Gunji and Nakamura, 2022a,b). Lattice theory has also been developed in programming and computation to analyze programming (Scott, 1972, 1976; Nielson et al., 2005).

#### 3.5.1. Definition of a lattice

A collection of elements that are distinct from each other is a set, but in a set, there is no relationship between the elements. In mathematics, a relationship is introduced between these elements to allow the structure to be considered. The simplest structure is an ordered set that introduces an order between elements (Davey and Priestley, 2002).

We first define an order. A subset of the direct product set is called a relation for the set *S*, and a relation that satisfies specific conditions is an order. The direct product of *S* is a set consisting of all pairs of elements of *S*. If *S* = {*a, b*}; then, we write the direct product of *S* as *S*×*S* = {(*a, a*), (*a, b*), (*b, a*), (*b, b*)}. A relation *R* is any subset of the product and is called the relation *R* on *S*. For example, as one of the relations of *S*×*S* = {(*a, a*), (*a, b*), (*b, a*), (*b, b*)}, *R* = {(*a, a*), (*a, b*), (*b, a*)}, which is a relation on *S* = {*a, b*}. If the element (*a, b*) is an element of the relation *R*, this is written as *aRb*. In the above example, we can write *aRa, aRb*, and *bRa*. A relation on *S* that satisfies conditions (1) to (3) below is called an ordered relation or simply an order. That is, for any *x, y, z*∈*S*,


(1) $$\begin{array}{l} xRx \end{array}$$

(2) $$\begin{array}{l} xRy\;and\;yRx\;\Rightarrow x=y \end{array}$$

(3) $$\begin{array}{l} xRy\;and\;yRz\;\Rightarrow xR \end{array}$$

An order is often represented by ≤. That is, *xRy* is written as *x* ≤ *y*. A set in which any elements satisfy (1) to (3) is called an ordered set.

For any two elements *x, y* of the ordered set *P*, the join of *x* and *y* is represented as *x*∨*y* and is defined by conditions (4) and (5).


(4) $$\begin{array}{l} x\leq x\vee y,\;y\leq x\vee y, \end{array}$$

(5) $$\begin{array}{l} x\leq z\;and\;y\leq z\;\Rightarrow \;x\vee y\leq z. \end{array}$$

Similarly, the meet operation represented by *x*∧*y* is defined by conditions (6) and (7).


(6) $$\begin{array}{l} x\wedge y\leq x,\;x\wedge y\leq y, \end{array}$$

(7) $$\begin{array}{l} z\leq x\;and\;z\leq y\;\Rightarrow \;z\leq x\wedge y. \end{array}$$

An ordered set *P* in which any two elements *x, y*∈*P*, *x*∨*y* and *x*∧*y* are also elements of *P* is called a lattice. In this case, join and meet can be considered (binary) operations similar to addition and multiplication. A lattice is an algebra in the sense that it is closed for this operation (the result of the operation is also an element of it).

**Figure 7:**
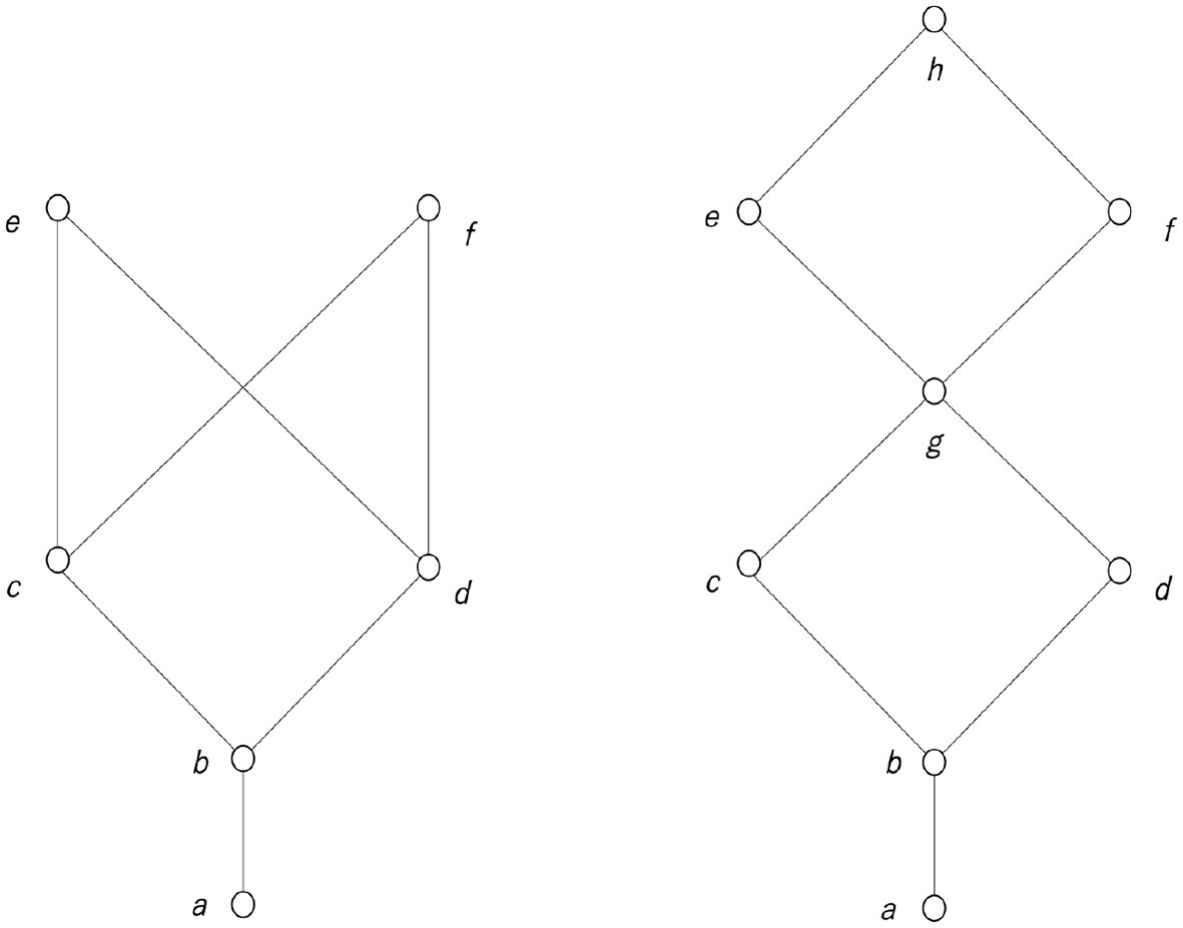
Non-lattice ordered set **(left)** and a lattice **(right)**.

Figure 7 is a diagram called a Hasse diagram that illustrates an ordered set. A Hasse diagram is used to illustrate the structure of a lattice. In the Hasse diagram, the elements are drawn in circles so that they do not overlap; if *x* ≤ *y*, the circle representing *y* is drawn above the circle representing *x*, and those two circles are connected by a line. However, if *x* ≤ *y*, and if there is another element *z* such as *x*<*z*<*y*, the circles representing *x* and *y* are not connected by a line. In Figure 7, the left Hasse diagram is not a lattice, although the right diagram is a lattice.

A lattice *L* is defined as a distributive lattice if and only if for any *x, y, z*∈*L, x*∧(*y*∨*z*) = (*x*∧*y*)∨(*x*∧*z*). A lattice *L* is defined as a complemented lattice if and only if for ∀*x*∈*L*, ∃*x*^*c*^∈*L* such that *x*∧*x*^*c*^ = 0 and *x*∨*x*^*c*^ = 1, where 0 and 1 represent the least and the greatest values of *L*, respectively. A Boolean lattice (algebra) is defined by a complemented distributive lattice (Davey and Priestley, 2002).

#### 3.5.2. A lattice whose elements are sets

**Figure 8:**
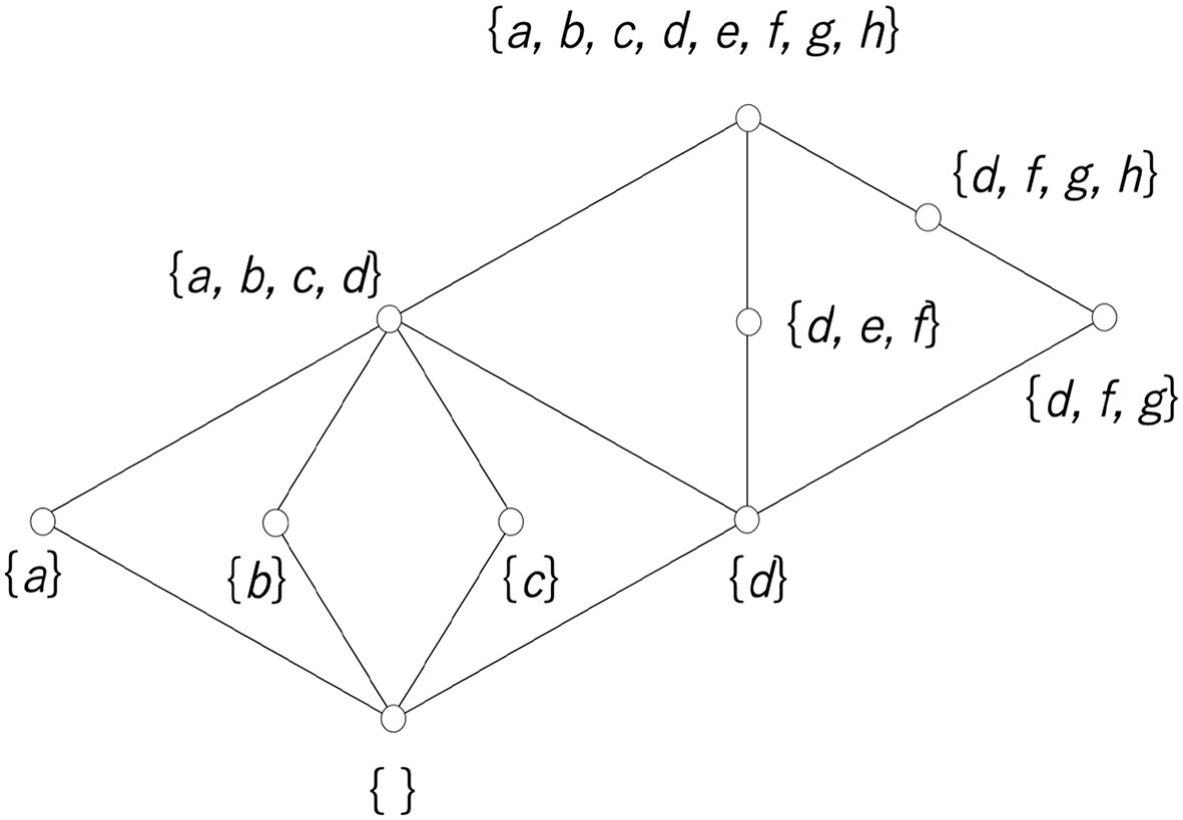
Example of a lattice whose elements are sets.

An element of the lattice used in the analysis of this study is a set of elements. When the elements are a set, the order relation is defined by the inclusion relation. If any element of set *A* is an element of set *B*, *A* is included in *B*, which is expressed by *A*⊆*B*. This inclusion relation clearly satisfies (i) *A*⊆*A*; (ii) *A*⊆*B* and ⊆*A* ⇒*A* = *B*; and (iii) *A*⊆*B* and *B*⊆*C* ⇒*A*⊆*C*, which correspond to conditions (1) to (3). The join and meet operations, *A*∨*B* and *A*∧*B*, are also defined, and they satisfy conditions (4) to (7). Figure 8 shows an example of a lattice whose elements are sets. The symbol {} with no elements indicates the empty set.

#### 3.5.3. Rough set lattice

When applying a rough set to a lattice, we use a map, kernel, and equivalence relation. Therefore, we first define these notions. A set is defined as a collection of elements that are distinct from each other. There is no structure other than discernibility. Given a pair of sets, a map is defined from one set, called the domain, to the other, called the codomain, such that for any element of the former set, there is a unique element in the latter set.

**Figure 9:**
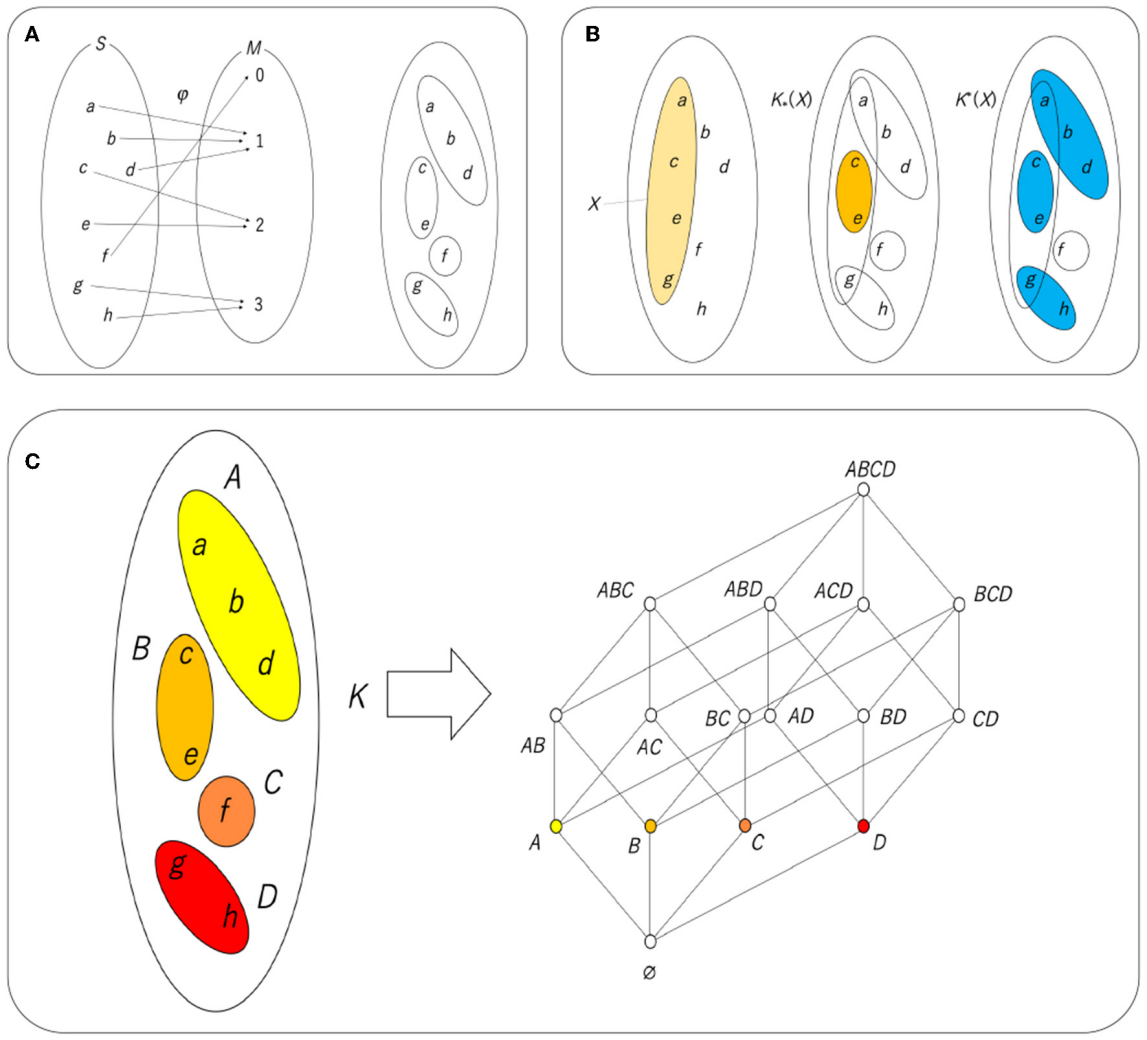
**(A)** Cognitive process φ and the equivalence class derived from it. **(B)** *X* (left), K_*_(*X*) (center), and K^*^(*X*). **(C)** Hasse diagram of a lattice resulting from $K^{*}\left(K_{*}\left(X\right)\right)=X$. An equivalence class with a certain color corresponds to an atom with the same color. Note that *AB* in the Hasse diagram is an abbreviation of *A*∪*B*.

Suppose that the sets *S* and *M* and a map from *S* to, φ, are given. Here, if *S* is interpreted as the set of real phenomena and *M* as the set of representations that are the result of cognition, the mapping φ is cognition. Let us assume that this cognitive process is defined as shown in Figure 9. Figure 9A shows that *a, b*, and *d*, which are distinct phenomena, are perceived as the same value, 1. To summarize all the cognitive situations, φ(*a*) = φ(*b*) = φ(*d*), φ(*c*) = φ(*e*), φ(*f*), φ(*g*) = φ(*h*). This means that *a, b*, and *d* are the same with respect to φ and are called the kernel of φ in universal algebra. Strictly speaking, the kernel is defined by Kerφ = {(*x, y*)∈*S*×*S*|φ(*x*) = φ(*y*)}. The kernel can allow an equivalence relation. Given a set *S*, a subset of *S*×*S* is called a relation. The relation *K*⊆*S*×*S* is an equivalence relation if and only if *K* satisfies, for any *x, y, z*∈*S*, (i) *xKx*, (ii) *xKy* ⇒*yKx*, and (iii) *xKy* and *yKz* ⇒*xKz*. Note that *xKy* implies (*x, y*)∈*K*. It is straightforwardly verified that Kerφ is an equivalence relation, and it is replaced by *K* here.

A mapping defines a destination for all the elements of a set of domains and is not allowed if there are multiple destinations for an element. Therefore, by mapping, the set of domains is divided into subsets that are disjoint (no overlap). Each of these divided groups is called an equivalence class (Figure 9A). Given an equivalence relation *K*, an equivalence class is defined by [*x*]_*K*_ = {*y*∈*S*|*xKy*}. In using this notation, [*a*]_*K*_ = {*a, b, d*}. The division using the obtained equivalence class as a unit can be said to be a set that is coarse-grained with respect to the set of original phenomena. In that sense, a set whose unit is this equivalence class is called a rough set. Fuzzy sets and fuzzy logic are known from the perspective of coarse-grained sets and the logic derived from them, but fuzzy sets aim for coarse-grained continuous quantities, and for that purpose, it is necessary to arbitrarily define the membership function. On the other hand, a rough set can derive all approximations from one map, and the method used in the phase structure can be used as it is (Pawlak, 1981, 1982; Polkowski, 2002). Therefore, in recent years, rough sets have been widely used in the field of soft computing instead of fuzzy sets.

The method used in the topological structure is “approximation.” Similar to considering a set of interior points or the closure of a set, it is possible to define lower and upper approximations for equivalence relations and perform approximate calculations. Figure 9B shows an example of lower approximation and upper approximation by the equivalence relation *K* obtained in Figure 9A. Here, *X* is given as a subset of the set *S*. If the recognizing agent can directly recognize all the elements of *X*, then *X* will be recognized as *X*. However, here, the agent can only recognize the equivalence class of *K* and can only recognize a subset of *S* as a combination of equivalence classes. In other words, *X* is approximated as a combination of equivalence classes. There are two types of approximation. The first is a lower approximation, represented as K_*_(*X*), which is a collection of elements in equivalence classes of *K* contained in *X*. The second is the upper approximation, represented as K^*^(*X*), which is a collection of equivalence classes of *K* having non-empty intersections with *X*. They are formally defined by the following equations:


(8) $$\begin{array}{l} K_{{}^{*}}\left(X\right)=\left\{x\in S|{\left[x\right]}_{K}\subseteq X\right\}, \end{array}$$

(9) $$\begin{array}{l} K^{{}^{*}}\left(X\right)=\left\{x\in S|{\left[x\right]}_{K}\cap X\neq \emptyset \right\}. \end{array}$$

In Figure 9B, K_*_(*X*), which is a union of the equivalence classes included in *X*, is represented by {*c, e*}, and K^*^(*X*), which is a union of the equivalence classes that have non-empty intersections with *X*, is represented by the union of {*a, b, d*}, {*c, e*}, and {*g, h*}, that is, {*a, b, c, d, e, g, h*}. By definition, K_*_(*X*) is contained in *X* and *X* is contained in K^*^(*X*), so K_*_(*X*) is a sufficient condition for *X*, and K^*^(*X*) is a necessary condition for *X*. In that sense,


(10) $$\begin{array}{l} K_{*}\left(K^{*}\left(X\right)\right)=X \end{array}$$

implies a necessary and sufficient condition for *X*. It is easy to see that any combination of equivalence classes can satisfy Equation (10), as shown in Figure 9C. This ordered set is a lattice since the meet of two elements is expressed as the intersection of the two elements and the join is expressed as the union; this is a Boolean lattice or Boolean algebra.

**Figure 10:**
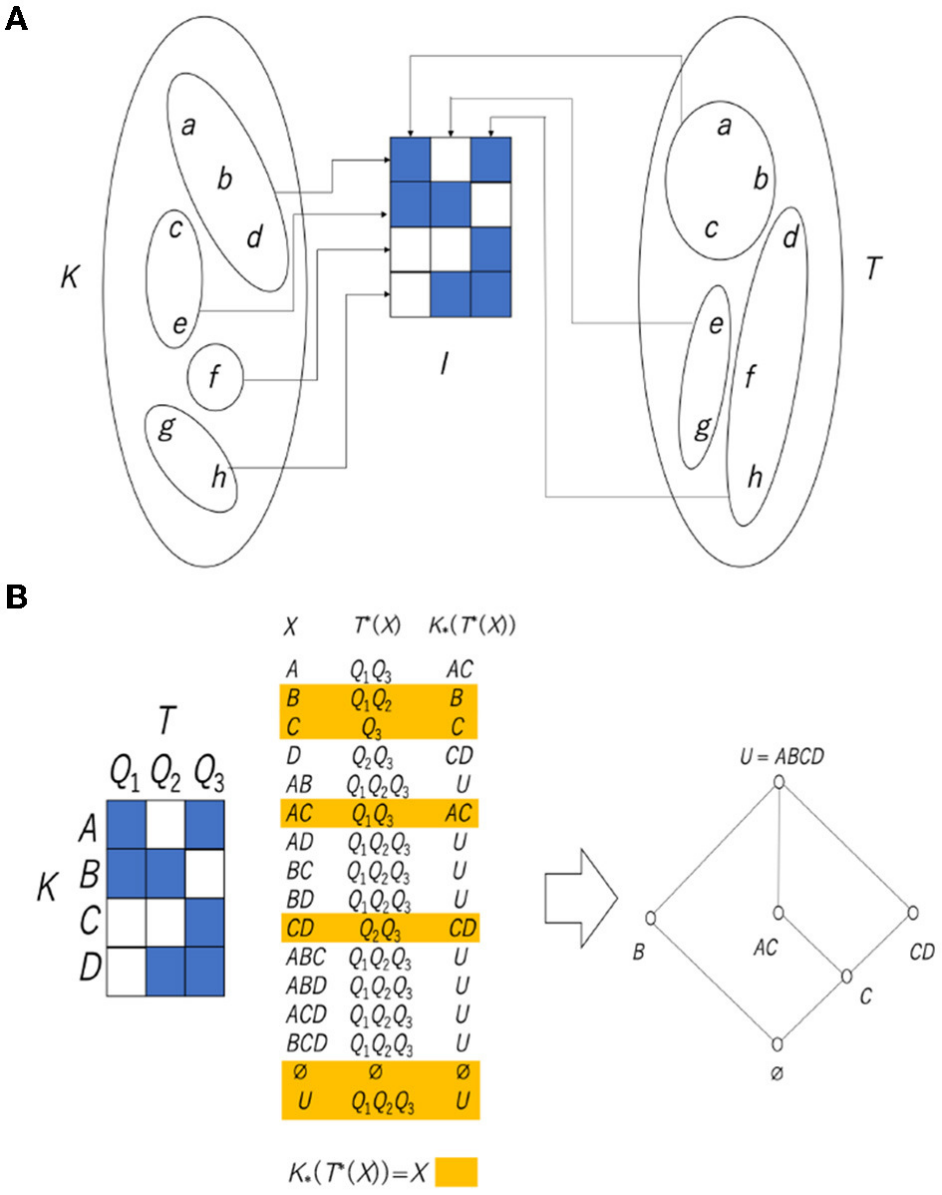
**(A)** Relation obtained from two types of cognition. **(B)** Rough set lattice derived from the relationship between two types of equivalence classes. Note that *AC* in the central table and the Hasse diagram is an abbreviation of *A*∪*C*.

There are two cognitive processes, namely visual and tactile, and it is thought that the way each phenomenon is received is different. Potatoes and pebbles look the same visually, but the difference can be determined by touching them. On the other hand, frog skin and the surface of jelly feel the same, but the difference is immediately visible. Since the cognition and perception of the phenomena differ depending on the sensory mode, it is concluded that the division of a world with such phenomena as elements differs depending on the sensory mode. Figure 10A illustrates this situation. It is assumed that the two divisions placed on the left and right are the divisions obtained from two different cognitive processes. Here, for convenience, let us assume that the left side is divided by sight (equivalence relation *K*) and the right is divided by tactile sensations (equivalence relation *T*). A relation *I* can be defined from these two divisions. In Figure 10A, the central 3 × 4 matrix takes four equivalence classes of visual sensation in the vertical direction and three equivalence classes of tactile sensation in the horizontal direction. If an equivalence class of *K* is represented by *x* and an equivalence class of *T* is represented by *y*, then *xIy* (presence of a relation; blue cell) is defined by common elements that exist in both *x* and *y*, and the absence of a relation (blank cell) is defined by no element existing in both *x* and *y*. For example, since a cell at (1, 2) indicates a pair of a visual equivalence class {*c, e*} and a tactile equivalence class {*a, b, c*}, there exists a common element, *c*, and this cell is colored blue.

In the case of one equivalence relation, the necessary and sufficient condition is given by $K_{*}\left(K^{*}\left(X\right)\right)=X$, but in the case of two equivalence relations, one equivalence relation is used as a necessary condition. By using the other as a sufficient condition, an equation that satisfies the necessary and sufficient conditions can be obtained as follows:


(11) $$\begin{array}{l} K_{*}\left(T^{*}\left(X\right)\right)=X. \end{array}$$

Including Equation (11), four types of necessary and sufficient conditions can be obtained, such as $T_{*}\left(K^{*}\left(X\right)\right)=X,\;K^{*}\left(T_{*}\left(X\right)\right)=X$, and $T^{*}\left(K_{*}\left(X\right)\right)=X,$ and it is known that the set of all *X* is a lattice and they are all isomorphic (structurally the same) to each other (Gunji and Haruna, 2010). Since the set obtained through the upper approximation and the lower approximation is at most the union of the equivalence classes, the lower approximation and the upper approximation can be calculated by considering only the compositions of the equivalence classes. The relationships obtained from the two equivalence classes directly implement this.

Figure 10B shows how to construct a rough set lattice derived from Equation (11). We assume that there is a relation *I* between a set of equivalence classes *A, B, C, D* with respect to the equivalence relation *K* and a set of equivalence classes *Q*_1_, *Q*_2_, *Q*_3_ with respect to the equivalence relation *T*. The central table shows the calculation of X, T^*^(*X*) and $K_{*}\left(T^{*}\left(X\right)\right)$. After that, we collect the *X* satisfying $K_{*}\left(T^{*}\left(X\right)\right)=X$ (highlighted in the table in Figure 10B), and we can obtain a rough set lattice, as shown in the right Hasse diagram in Figure 10B.

#### 3.5.4. Analysis in this study

In this study, we consider representations and objects as two types of equivalence classes, obtain a relationship between them using the results of subjective questionnaires, and obtain a rough set induction lattice from them. We evaluate the algebraic structure from the structure of the lattice.

## 4. Results

### 4.1. Subjective task

From the preexperimental questionnaire, regarding factors such as gender, age, dominant hand, and sports history, no significant differences were found.

**Figure 11:**
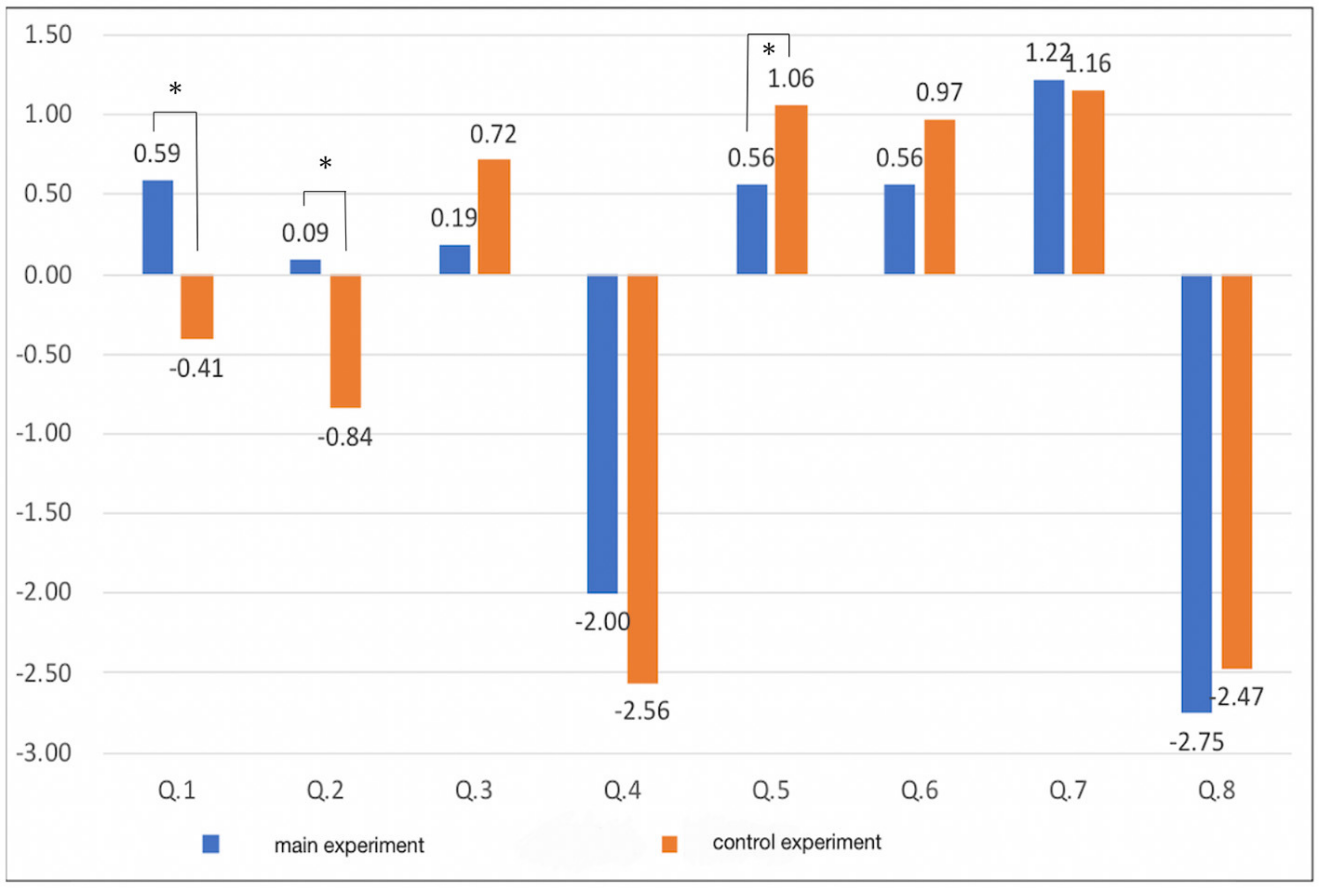
Subjective questionnaires and averages for each question. *p < 0.05.

The results of the questionnaire after performing the subjective task are shown in Table 6 and Figure 11. In Q1 and Q2, the difference in the subjective intensity of the main experiment with respect to the control experiment is larger than that of the other questions. The subjects could feel as though the thumb had disappeared, and the illusion of a four-fingered hand was induced when the monkey's hand was held. Additionally, when viewed individually, the main experiment had stronger results than the control experiment in Q1. There were 17 people who had more intense feelings, 10 people with the same intensity, and five people who had less intense feelings. In Q2, the main experiment had stronger results than the control experiment. There were 18 people who had more intense feelings, seven people with the same intensity, and seven people who had less intense feelings. There were seven answers that did not fit the survey intention, but when the subjects were asked the reason for each answer, they gave responses such as “I didn't know which one was the index finger and it was like I had no index finger (main Experiment Q4)” and “Since the first part of the thumb (trapezium) was visible, it was interpreted as the thumb (control Experiment Q8)”; therefore, we did not exclude the data of these respondents.

**Table 6:** Comparison of average values and *t*-tests for each question in the subjective questionnaire.

**No**.	**Contents of question**	**Average value (main experiment)**	**Average value (control experiment)**	**Difference in means**	***p*-value (two-sided *t*-test)**
Q1	Because it looks like a four-fingered hand, I realize that I don't have a thumb	0.61	−0.45	1.06	0.006
Q2	I feel that my thumb doesn't exist	0.06	−0.84	0.90	0.027
Q3	Being able to see the middle finger has nothing to do with being able to see the other fingers	0.23	0.77	−0.55	0.071
Q4	I feel that the index finger does not exist	−2.10	−2.58	0.48	0.053
Q5	Seeing the ring finger is sufficient to notice the existence of the ring finger	0.65	1.13	−0.48	0.047
Q6	Fingers other than the visible index finger are independent of seeing the index finger	0.58	0.94	−0.35	0.152
Q7	The existence of the little finger is noticed only by seeing the little finger	1.32	1.13	0.19	0.837
Q8	I can see my thumb	−2.74	−2.45	−0.29	0.095

A *t*-test was performed on the mean values of the experiment and the control experiment. The results are shown in the rightmost row of Table 6. Q1 has a *p*-value of ≈ 0.006 < 0.05, Q2 has a *p*-value of ≈ 0.027 < 0.05, Q5 has a *p*-value of ≒ 0.047 < 0.05, and it can be said that there is a significant difference between them.

### 4.2. Experiment 1

**Figure 12:**
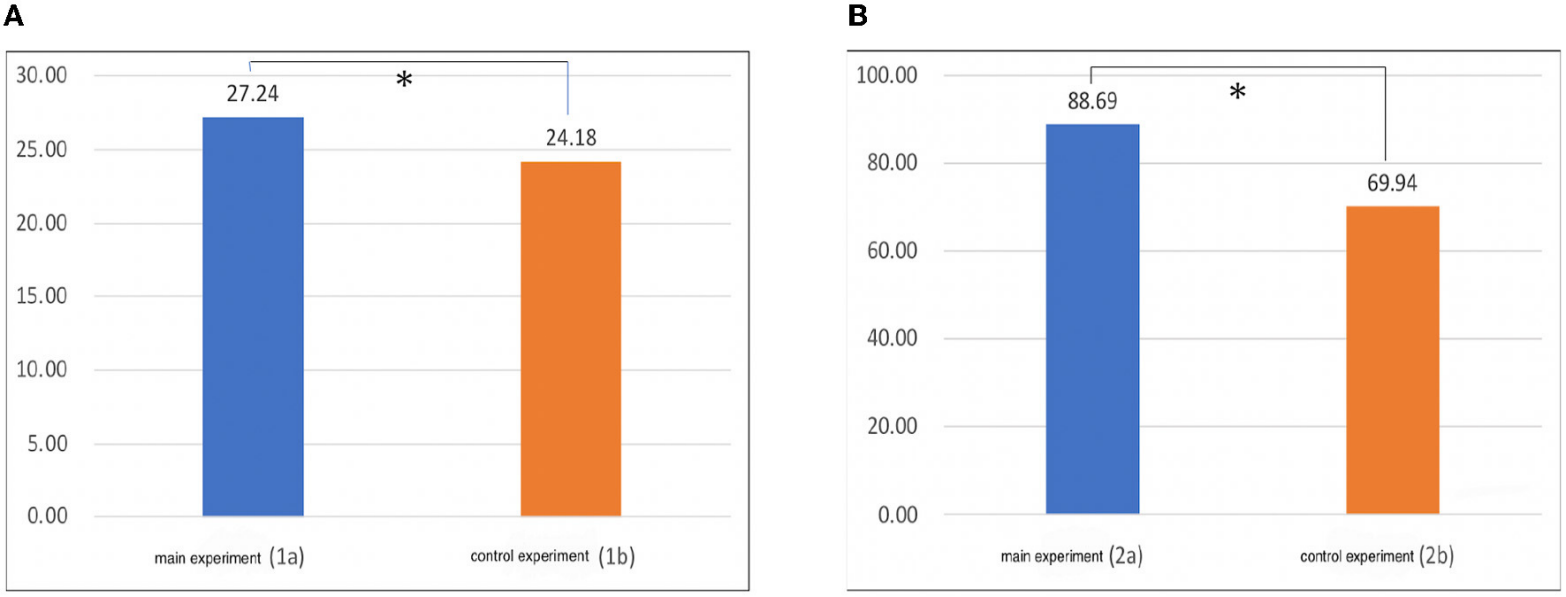
**(A)** Results of Experiment 1. **(B)** Results of Experiment 2. The vertical axis represents the drift (mm). *p < 0.05.

In Experiments 1a (main experiment; open eyes condition) and 1b (control experiment; closed eyes condition), the total deviation distance [mm] between the position that was actually touched and the position identified by the subject was normalized by dividing it by the length of the subject's nail (the length of the center of the nail). The value of Experiment 1a was 27.24, and the value of 1b was 24.18 (Figure 12A). When the significance of the difference was examined using the *t*-test, a *p*-value of ≒ 0.023 < 0.05 was obtained, and it was found that there was a significant difference.

From this result (Figure 12), we conclude that the deviation could be felt as larger when looking at the four-fingered hand than when the eyes were closed. Additionally, when viewed individually, 1a is more misaligned than 1b. There were 21 people with a large total value and 11 people with a small total value.

### 4.3. Experiment 2

In Experiments 2a (main experiment; open eyes condition) and 2b (control experiment; open eyes condition), the total distance [mm] between the tip of the index finger of the right hand and the tip of the thumb of the left hand at the position where the subject stopped was calculated. The graph in Figure 12B shows the average value for all subjects. The value of Experiment 2a was 88.69, and the value of 2b was 69.94. When the significance of the difference was examined using the *t*-test, a *p*-value of ≒ 0.006 < 0.05 was obtained, and it was found that there was a significant difference. From this result, as in Experiment 1, the deviation was larger when looking at the four-fingered hand than when the eyes were closed. When viewed individually, 20 people had a larger total deviation than 2b in 2a, and 12 people had a smaller deviation.

### 4.4. Correlation between the experimental values and subjective reports

**Figure 13:**
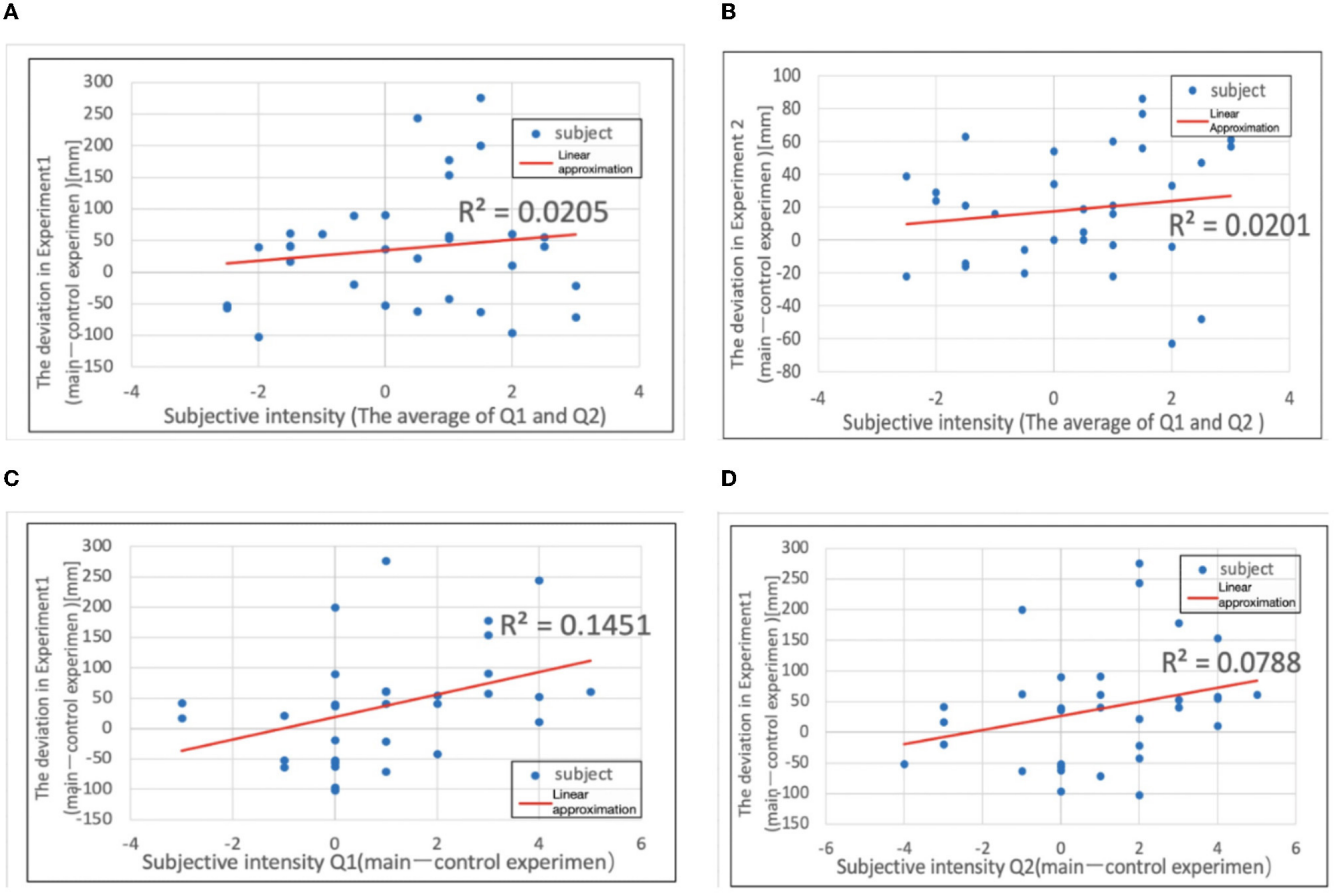
**(A)** Deviation in Experiment 1 plotted against subjective intensity. **(B)** Deviation in Experiment 2. **(C)** Deviation in Experiment 1 plotted against subjective intensity (Q1). **(D)** Deviation in Experiment 2 plotted against subjective intensity.

It was investigated whether there was a correlation between the intensity of the subjective illusion that the thumb was lost and the magnitude of the deviation in Experiments 1 and 2. The formula used in the correlation analysis is as follows. The correlation between Experiment 1 and the subjective questionnaire (this experiment; Q1, Q2 average) is plotted as *y* against *x*, where *x* = {[subjective questionnaire Q1 (main experiment)] + [subjective questionnaire Q2 (main experiment)]}/2 and *y* = [total value in Experiment 1a (mm)] – [total value in Experiment 1b (mm)]. The correlation between Experiment 2 and the subjective questionnaire (this experiment; Q1, Q2 average) is plotted as *y* against *x*, where *x* = {[subjective questionnaire Q1 (main experiment)] + [subjective questionnaire Q2 (main experiment)]}/2 and *y*= [total value in Experiment 2a (mm)] – [total value in Experiment 2b (mm)]. Note that Q1 is “Because it looks like a four-fingered hand, I realize that I don't have a thumb” and Q2: “I feel like my thumb doesn't exist.” Figures 13A, B shows the results of the analysis using the above formula.

When the approximate curves were calculated, the slope of Experiment 1 was 8.285, and the slope of Experiment 2 was 3.129, both showing gentle positive slopes. However, from the correlation analysis, the correlation coefficient was *r* ≒ 0.143 for Experiment 1 and *r* ≒ 0.142 for Experiment 2, and there was almost no correlation. Furthermore, when the correlation with Experiment 1 was examined separately for the subjective questionnaires Q1 and Q2, a stronger correlation was obtained than before. The correlation between Experiment 1 and subjective questionnaire Q1 is plotted as *y* against *x*, where *x* = [subjective questionnaire Q1 (main experiment)] [subjective questionnaire Q1 (control experiment)] and *y* = [total value in Experiment 1a (mm)] [total value in Experiment 1b (mm)]. The correlation between Experiment 1 and subjective questionnaire Q2 is also plotted as *y* against *x*, where *x* = [subjective questionnaire Q2 (main experiment)] – [subjective questionnaire Q2 (control experiment)] and *y* = [total value in Experiment 1a (mm)] – [total value in Experiment 1b (mm)]. Figures 13C, D shows the results of the analysis using the above formula.

When the approximate curves were calculated (Figure 13), the slope of Experiment 1 was 18.57, and the slope of Experiment 2 was 11.58, both showing positive slopes. Furthermore, from the correlation analysis, Experiment 1 had *r* ≈ 0.381. In Experiment 2, *r* ≈ 0.281, and it was found that both had a low correlation. We investigated Experiment 2 in the same way, but there was no correlation (Experiment 2 and subjective Q1: *r* = 0.118, Experiment 2 and subjective Q2: *r* = 0.184).

### 4.5. Postexperiment questionnaire

Table 7 below shows the results of the postexperiment questionnaire (normal monkey hand position) conducted after Experiment 1 and the postexperiment questionnaire (reversed monkey hand position) conducted after all experiments were completed. The purpose of this experiment was to investigate how the subjects felt while performing the experimental task, but as seen from the comparison in each question, the intensity of the illusion of having four fingers was greater in the main questionnaire.

**Table 7:** Comparison of mean values and *t*-tests for each question in the postexperimental questionnaire.

**No**.	**Contents of question**	**Average value (normal monkey hand)**	**Average value (reversed monkey hand)**	**Difference in means**	***p*-value (two-sided *t*-test)**
Q1	After moving my four fingers freely, I felt like my thumb wasn't there	0.03	−1.56	1.59	0.0003
Q2	I felt that the hand was originally a four-fingered palm, rather than a five-fingered hand with the thumb missing	−0.28	−1.84	1.56	0.0001
Q3	I felt that the four-fingered palm was my palm.	1.50	0.53	0.97	0.0393
Q4	I felt that my four fingers were free to move	2.13	1.69	0.44	0.2250
Q5	I felt that the arm with the palm and four fingers was newly added	−2.28	−2.41	0.13	0.3795
Q6	I felt that my invisible thumb had moved somewhere else on my body	−2.13	−2.38	0.25	0.3399
Q7	I felt that the skin of my four fingers had a different texture than my palms	−1.03	−1.44	0.41	0.3525
Q8	I felt that my palm was covered with a four-fingered palm	−1.97	−1.97	0.00	1.0000
Q9	In Experiment 1, I felt that I was touching something other than my own hands	−1.78			
Q10	I felt pain when I touched my hand in Experiment 1	−2.88			

However, few respondents answered “applicable” to the question asking whether there was an extremely strong illusion in Q5–Q9 (10.8% of the respondents answered “1” or higher in Q5–Q9). Q9 and Q10 can only be answered by a subject in the normal monkey hand position, so they are omitted from the questions for the reversed monkey hand position. Q1 has a *p*-value of ≒ 0.0003 < 0.05, Q2 has a *p*-value of ≒ 0.001 < 0.05, and Q5 has a *p*-value of ≒ 0.0393 < 0.05; there is a significant difference between them.

### 4.6. Recognition of the absence of the thumb and its corresponding lattice

Here, the results of the “monkey hand” condition and the “hidden thumb hand” condition in the subjective report questionnaire are analyzed with respect to a lattice structure, which is an algebraic structure. The structure is clarified, especially the significance of the hidden thumb.

First, let us describe how to express the relationship between an object and its representation. Here, instead of recognizing and representing the so-called “raw” object, we consider that the object is also an equivalence class that targets the “raw” phenomenon. Even when one identifies an animal as a cat, “cat” is not the name of a specific object but the name of a set consisting of various concrete individuals, such as tabby cats and black cats. This is nothing but an equivalence class, all elements of which are equivalent with respect to the character of the cat.

**Figure 14:**
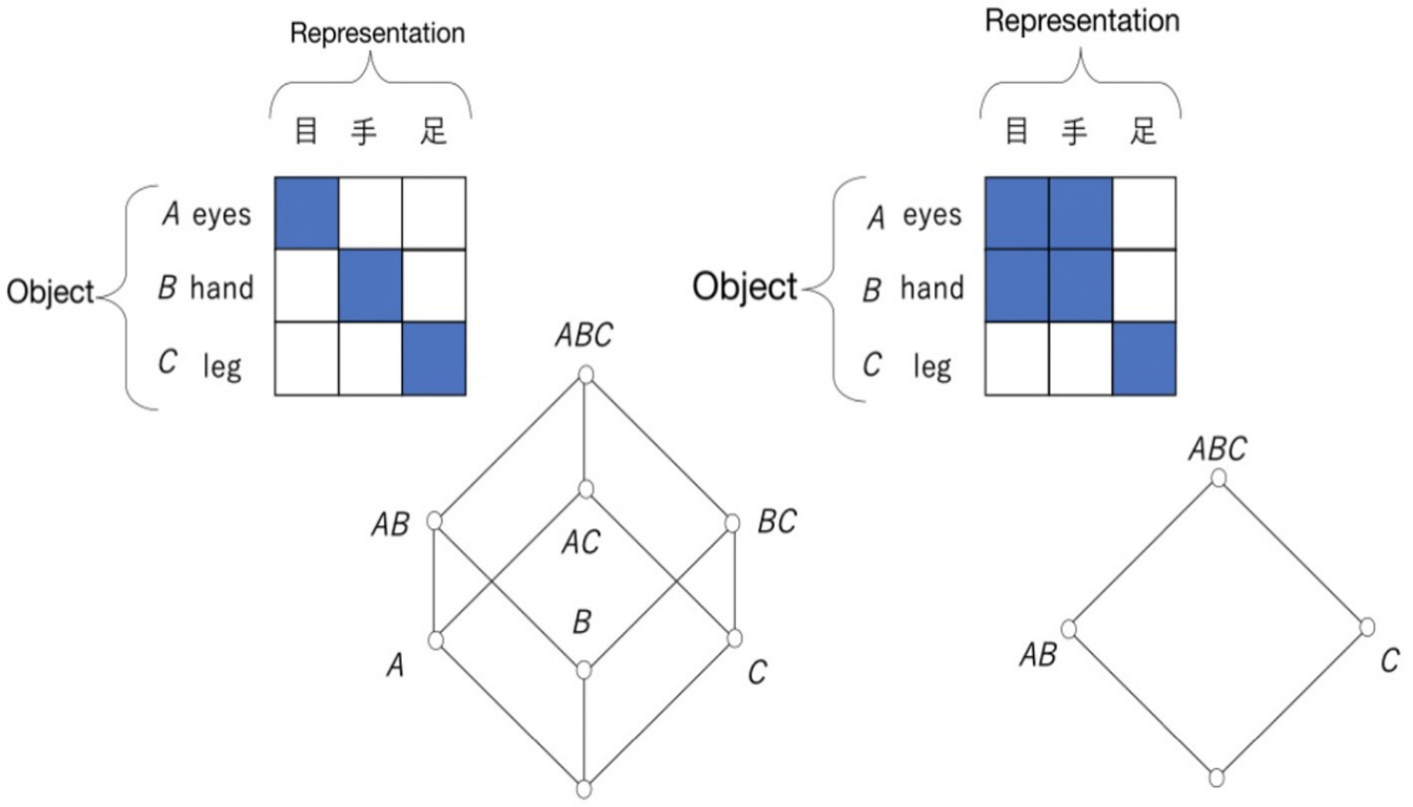
Two relations between objects and representations and their corresponding rough set lattices.

Here, the object is an element of the world that is recognized as an individual, and the representation is a system element that is forced to have a relationship with the other elements as a system. Additionally, the object and the representation consist of the same element, and it is assumed that the relationship between them is symmetric. The meaning of symmetry is shown in Figure 14. Here, only the hands, feet, and eyes are considered; the representation is in Chinese characters, Kanji, and the object name is written in English. There is a relationship between the object and the representation when the object to be represented exists. In that sense, if an object exists and is recognized in each representation, the relationship exists only in the diagonal components of the relation (Figure 14, upper left diagram). The corresponding rough set lattice is shown in the lower left figure of Figure 14. Note that this is also a Boolean algebra. Of course, if we try to represent a hand but it is lost in an accident, there is no relationship between the representation of the hand and the object. Such a case is possible. Figure 14, upper right diagram, shows that the eye to be represented is related not only to the eye as the object but also to the hand as the object. In this case, from the assumption of symmetry, the eyes as objects are also related to both the eyes and hands as representations. This means that the eyes and hands work together, for example, when playing table tennis, and the hands can react quickly to visual information. In the case of Figure 14, upper right diagram, the legs are isolated. Symmetry also means that relationships between different parts (equivalence classes) of representations are possible only through the object, which allows the objects to be interlocked. Therefore, the relationship between objects is realized symmetrically through the representation. The rough set lattice obtained from the relationship between the eyes and hands is shown in the lower right diagram of Figure 14. This is also a Boolean algebra.

Using this relationship between objects and representations, we analyze the “monkey's hand” and “hidden thumb hand.” There is a statistically significant difference between affirming and denying the question content only in Q1 and Q2 between the “monkey hand” condition and the “hidden thumb hand” condition. There is a significant difference in Q5, but both are affirmed under the two conditions, and there is no difference between affirmation and denial, that is, in the “monkey hand” state:

Attribute 1: “Visible four-fingered hands” and “absence of a thumb” coexist;
Attribute 2: The thumb does not exist;
Attribute 3: Each of the four fingers other than the thumb can be recognized independently of the other fingers.

As mentioned in Sections 1 and 2, we concentrate on “discomfort” in body ownership, which leads to the feeling of the monkey hand. Since the monkey hand carries an ambiguous feeling of the presence of the thumb (in terms of structure) and absence of the thumb (in terms of function), we choose an attribute to abstract that ambiguous feeling. We prepared the subjective questionnaire to manifest this. Since Q1, “Because it looks like a four-fingered hand, I realize that I don't have a thumb” is positive and significantly different from the result of the control experiment, we can choose Attribute 1. Since Q2, “I feel that my thumb doesn't exist,” is positive and significantly different from the result of the control experiment, we can choose Attribute 2. Finally, the results for questions Q3, 5, 6, and 7 are positive, and they lead to the independence of the four fingers, that is, Attribute 3.

**Figure 15:**
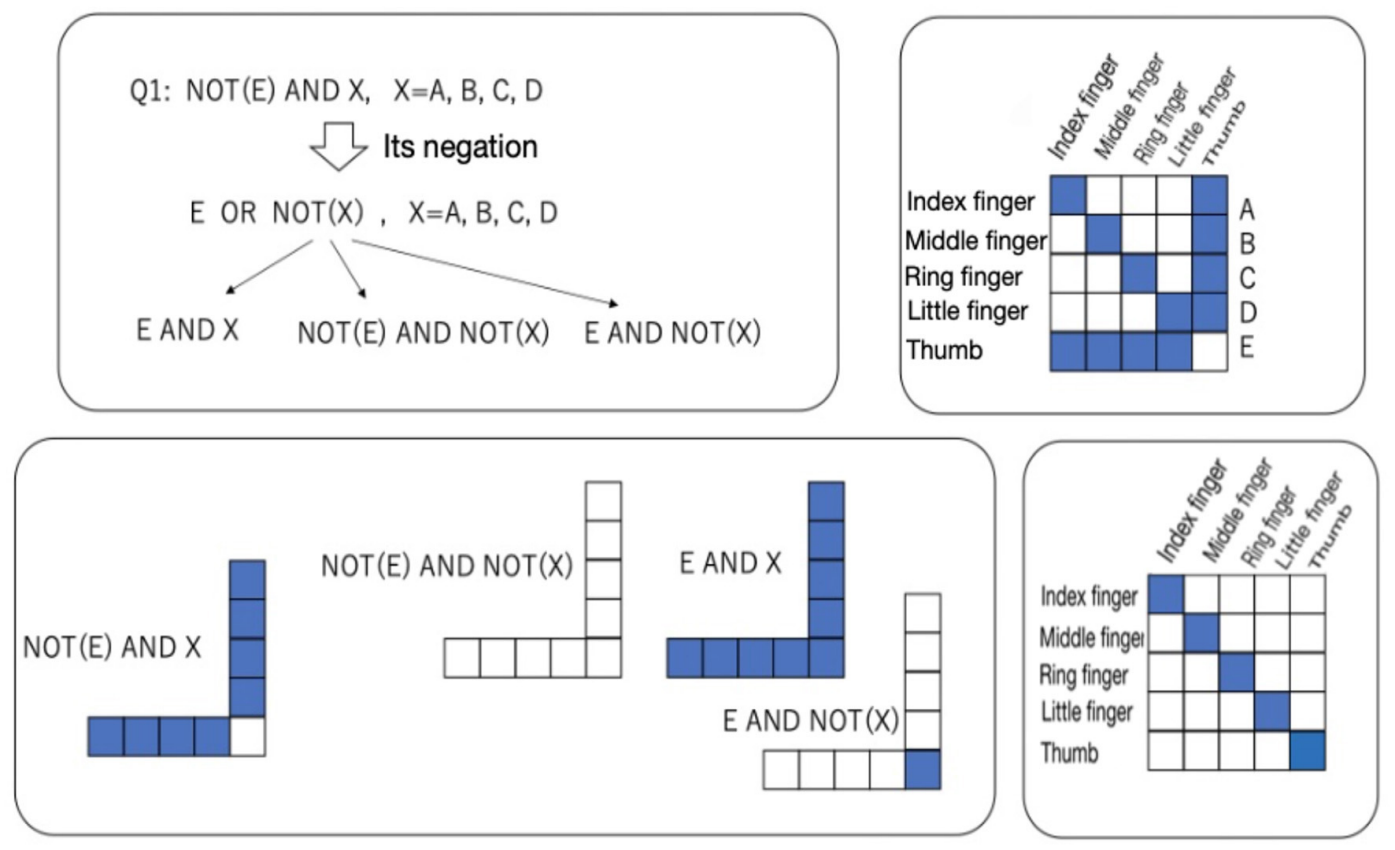
Affirmation and negation of Q1 **(upper left)** and their relations **(lower left)**; a relation between representations and objects satisfying Attributes 1 to 3 **(upper right)**; and a relation satisfying Attributes 1′-3, 2′, and 3′ **(lower right)**.

In Attribute 1, the condition “absence of a thumb” implies not simply that the thumb does not exist but that there is a feeling of not having a thumb that should exist. This implies that the non-existent thumb can be recognized as an illusion and that the illusion can be related to the other fingers. In contrast, Attribute 2 simply implies that the thumb as represented in the brain does not exist as an object and that an object that should be in the hand is not represented in the brain. The diagram in the upper right inner square in Figure 15 shows Attributes 1–3 as relations between objects and representations. The blank cell (i.e., no relation) in the relation at (Thumb, Thumb) simply implies Attribute 2. The blue cells (i.e., presence of a relation) in the relation at (Index finger, Thumb), (Middle finger, Thumb), (Ring finger, Thumb), and (Little finger, Thumb) and the blank cell at (Thumb, Thumb) imply a coexisting relation between the index, middle, ring, and little finger and the absence of the thumb, which implies Attribute 2. The assumption of a symmetry relation is expressed as the blue cells at (Thumb, Index finger), (Thumb, Middle finger), (Thumb, Ring finger), and (Thumb, Little finger, Thumb), and the blank cell at (Thumb, Thumb). Attribute 3 is expressed as the blue cells that are found only at the diagonal cells for the fingers other than the thumb. This implies that the fingers other than the thumb exist without a relation to other fingers (i.e., they are recognized independently of the other fingers).

On the other hand, Attributes 1 and 2 are denied under the “hidden thumb hand” condition. Since “visible four-fingered hands” and “absence of a thumb” are denied, one obtains the following:

Not (“visible four-fingered hand” and “absence of a thumb”)
= (Not “visible four-fingered hand”) or (not “absence of a thumb”)
= “I cannot see my four-fingered hand” or “I have a thumb.”

The statement “*A* or *B*” implies “only *A* is established, only *B* is established, or both *A* and *B* are established.” Therefore, denial of Attribute 1 in the “monkey's hand” condition is divided into three attributes in the “hidden thumb hand” condition, such as Attributes 1′-1, 1′-2, and 1′-3. Other attributes corresponding to Attributes 2 and 3 in the “monkey's hand” condition are expressed as Attributes 2′ and 3′ in the “hidden thumb hand” condition. Under the “hidden thumb hand” condition, the following attributes are obtained:

Attribute 1′-1: “I cannot see the four-fingered hand” + “I do not have a thumb”
Attribute 1′-2: “I can see the hands of four fingers” + “There is a thumb”
Attribute 1′-3: “I cannot see the four-fingered hand” + “There is a thumb”
Attribute 2′: There is a thumb.
Attribute 3′: Each of the four fingers other than the thumb can be recognized independently of the other fingers (the recognition of each finger is independent).

The negation of Attribute 1 leading to Attributes 1′-1 to 1′-3 mentioned above is shown as a diagram in the upper left inner square in Figure 15. The relations representing Attributes 1′-1 to 1′-3 are shown as the diagrams in the lower left inner square in Figure 15, where E and X represent Attribute 1′-2, NOT(*E*) and NOT(*X*) represent Attribute 1′-1, and *E* and NOT(*X*) represent Attribute 1′-3 under the “hidden thumb hand” condition. The relationship between objects and the representation corresponding to each attribute is expressed as a relation in the lower left inner square in Figure 15. In contrast to the relation in the “monkey's hand” condition, the “normal hand” in which all fingers including the thumb are recognized independently is given as a diagonal relation in the lower right inner square in Figure 15.

**Figure 16:**
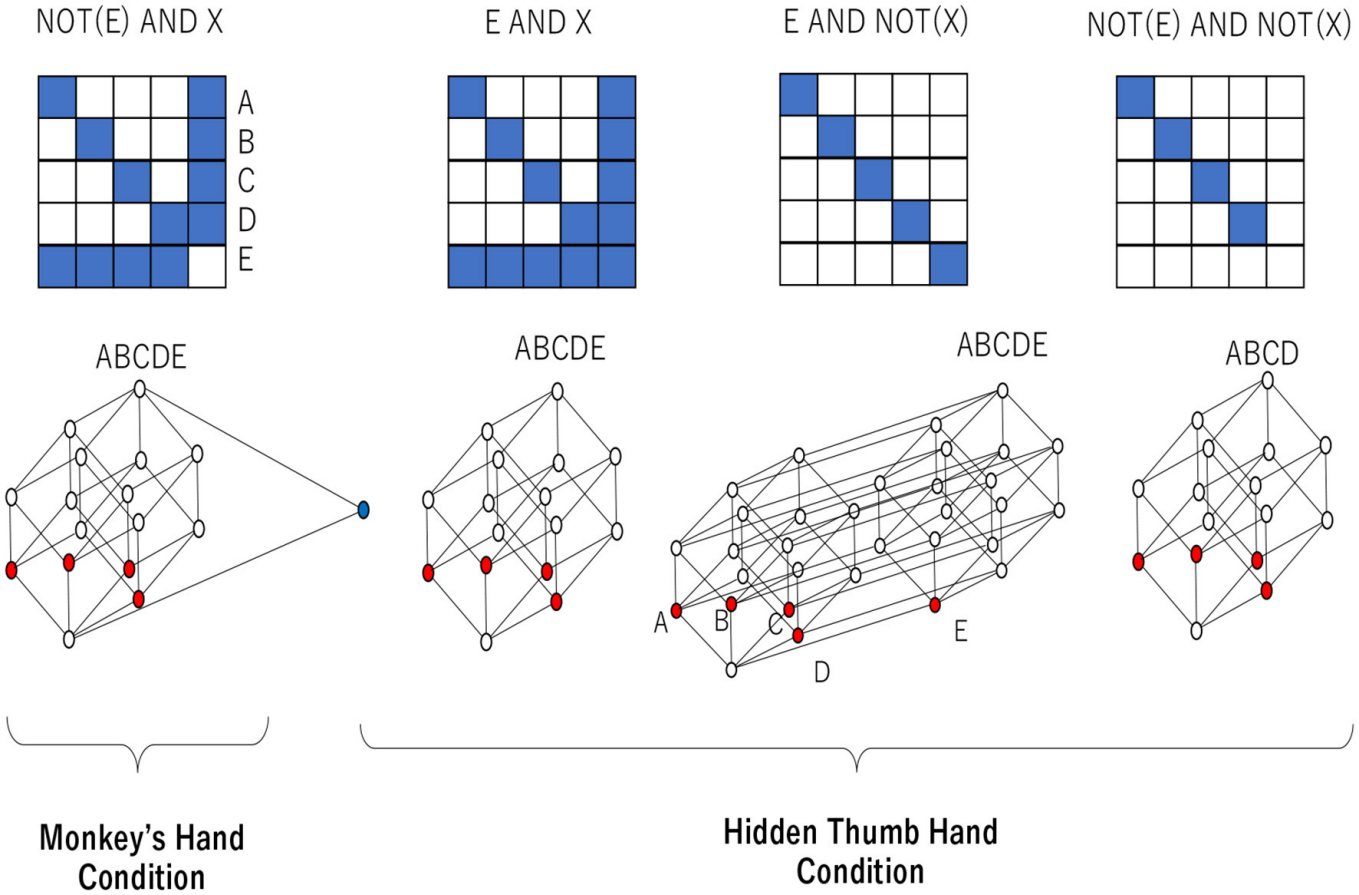
Representation/object relations and corresponding lattices in the “monkey's hand” and “hidden thumb hand” conditions.

From the above, considering all the attributes, the object–representation relation and the rough set lattice, as shown in Figure 16, can be obtained in the “monkey's hand” and “hidden thumb hand” conditions. The relation at the left end of Figure 16 represents Attributes 1 to 3 under the “monkey hand” condition, and the Hasse diagram below it shows the rough set lattice obtained from that relation. The three conditions on the right are a combination of Attributes 1′-1 to 1′-3 and Attribute 3′, but Attribute 2′ is not added, and they are recognized under the “hidden thumb hand” condition. All of them have different numbers of elements, but the number of equivalence classes, which is the basic unit of recognition, is different from 4 and 5, and they all cover all combinations of equivalence classes. This implies that any phenomenon of fingers is reducible to a single finger. On the other hand, in the lattice of the “monkey hand” condition, the four fingers other than the thumb form a Boolean algebra, and the thumb that is supposed to exist is “absent.”

One can check the rough set lattice corresponding to NOT(*E*) AND *X* in Figure 16 as follows. In calculating $K_{*}\left(T^{*}\left(X\right)\right)=X,\;$we assume that the equivalence classes of *T* and *K* are assigned with the same symbols and the same order, such as *A* (little finger), *B* (ring finger), *C* (middle finger), *D* (index finger), and *E* (thumb), and that the union of equivalence classes is represented by removing the union symbol, for example, representing *A*∪*B* as *AB*. If *X* = *A*, then $K_{*}\left(T^{*}\left(A\right)\right)=K_{*}\left(AE\right)=A$. This implies that *A* is an element of a rough set lattice. It is easy to see that any other fingers, including the thumb, can be an element of a rough set lattice since $K_{*}\left(T^{*}\left(D\right)\right)=K_{*}\left(DE\right)=D$ and $K_{*}\left(T^{*}\left(E\right)\right)=K_{*}\left(ABCD\right)=E$. It is also easy to check that any other combination of fingers except the thumb can be an element of a rough set lattice since $K_{*}\left(T^{*}\left(AB\right)\right)=K_{*}\left(ABE\right)=AB$ and $K_{*}\left(T^{*}\left(ACD\right)\right)=K_{*}\left(ACDE\right)=ACD$. However, the combination of equivalence classes containing the thumb (*E*) is not an element of a rough set lattice since $K_{*}\left(T^{*}\left(AE\right)\right)=K_{*}\left(ABCDE\right)=ABCDE$ and therefore $K_{*}\left(T^{*}\left(AE\right)\right)\neq AE$. Finally, one can obtain a set of all elements of the rough set lattice as {∅, *A, B, C, D, E, AB, AC, AD, BC, BD, CD, ABC, ABD, ACD*, } {*BCD, ABCDE*}. In contrast, the rough set lattice corresponding to *E* AND *X* in Figure 16 never contains the thumb (*E*) as an element since $K_{*}\left(T^{*}\left(E\right)\right)=K_{*}\left(ABCDE\right)=ABCDE$. In the Hasse diagram, elements of a lattice are linked to other elements by a line, which implies an inclusion relation.

In the Hasse diagram corresponding to NOT(*E*) AND *X*, all fingers except the thumb are represented by red circles. This implies that the little, index, middle, and ring fingers are recognized as basic units of recognition (i.e., atoms) and are recognized independently. The elements above the atoms are linked to the atoms, which shows that any combination of atoms can be recognized. On the other hand, the thumb is represented by a blue circle, which intersects only the least and the greatest elements of the lattice. There is no element between the least element and the thumb (blue circle), which implies that the thumb is also a basic unit of recognition. There is no element between the greatest element and the thumb (blue circle), which implies that the thumb cannot form combinations with any other finger. Thus, the lattice structure shows that the thumb is isolated from the other fingers. If the thumb is recognized as a real object, it can entail any component of any other finger, and vice versa. Therefore, if there is no possibility of combinations with the thumb, this implies that the thumb is recognized as an illusion. This implies that the thumb virtually exists but does not truly exist. This is an intrinsic property of the monkey's hand condition, showing the ambiguity of absence and presence.

In the Hasse diagram corresponding to *E* AND *X*, all fingers except the thumb are represented by red circles and are recognized as basic units of recognition. They are recognized independently, and any combination is possible. Although the thumb is not recognized independently, it is recognized that the thumb is contained in the set of all fingers. Thus, in this situation, the thumb is simply forgotten. In the Hasse diagram corresponding to *E* AND NOT(*X*), all fingers, including the thumb, are represented by red circles and are recognized as basic units of recognition. This is the same as the normal situation in which any finger can be recognized independently. In the Hasse diagram corresponding to NOT(*E*) AND NOT(*X*), all fingers except the thumb are represented by red circles and are recognized independently. It is clear that the thumb (*E*) is not contained in the set of all fingers. This implies that the thumb is recognized as lost.

Through a rough set lattice analysis of the subjective questionnaires, we obtain the essential difference between the “monkey's hand” condition and the “hidden thumb hand” condition. Under the hidden thumb hand condition, regardless of whether the thumb is recognized, any event can be recognized as a combination of basic units of recognition (i.e., atoms). In contrast, the thumb in the monkey's hand condition has a special status in which the thumb can be recognized but cannot be combined with any other fingers. This implies that the thumb is not lost and is recognized but is recognized as an illusion. What does one consider such an illusionary thumb? As a real object, it is recognized that four fingers without the thumb can constitute a whole hand and that the absence of the thumb does not imply that the thumb is something that should exist. In other words, the absence of the thumb is not recognized as a case of a missing thumb. This specific status of the thumb implies that the monkey's hand is naturally recognized as whole, with no parts missing. The monkey hand is passively accepted as a naturally deformed hand.

## 5. Discussion

In our monkey's hand experiment, the subjects sometimes felt discomfort. The results of Experiments 1 and 2 show that the palm of the subject's hand is expanded outward, which leads to the thumb being far from the other four fingers. This results in discomfort in the subject's thumb, since the thumb is located in a peculiar position, as if the hand were a monkey's hand. Therefore, the subjects feel as if the thumb is functionally lost and structurally present. This leads to the ambiguous feeling that the thumb is lost and present somewhere. Such ambiguity in body ownership reflects a lattice structure in which the thumb exists somewhere in the whole structure but the thumb has no relation to the other four fingers.

Our estimation of drift is different from the drift measurement in the rubber hand illusion. Compared to the drift in the rubber hand illusion, which is the distance between the subject's real hand and the rubber hand, the drift in our experiment has no pair of real and fake hands because the illusion is acquired only for a subject's own real hand. In this condition, the drift in our experiment measures the deformation of the subject's own real hand. Although the control experiment also makes the subject unable to see his or her own thumb, this condition never leads to the illusion of the deformation of the palm and thereby to the illusion of the monkey's hand. It does not result in the illusion of the ambiguity of the thumb in terms of (structural) presence and (functional) absence.

First, we will discuss the results of the subjective questionnaire. As seen from result 4-1, when looking at the hand in the monkey's hand condition, it feels as if one's hand has four fingers. In other words, it is possible to feel the transformational sensation that a part of one's body is absent only by a visual illusion, while the sense of ownership (SoO) is maintained. Typical impressions of the subjects regarding the subjective questionnaire include the following: “I had a strong feeling of not pointing at four,” “I felt the illusion of the monkey's hand,” and “I gradually began to accept the state of having no thumb in the subjective task.” It was concluded that after looking at the monkey's hand for some time, the subject felt that his or her thumb was originally absent and was not lost and that the condition was accepted as his or her own body image.

Next, we discuss proprioceptive drift as objective data. From Experiment 1, one can estimate the deformation of the surface of the palm as a whole. On the other hand, from Experiment 2, one can estimate the location of the thumb itself. While there was a possibility of thumb extension alone without deformation of the palm, the results show that the palm was expanded outward, which led to an extension of the thumb.

Experiments 1 and 2 evaluated how proprioceptive sensations are affected by the illusion. The following hypothesis was established. When looking at the “monkey's hand,” the visual stimulus is predominant due to the illusion, the proprioceptive sensation is weakened, and the position recognition shift is larger. In contrast, when the eyes are closed, it is hypothesized that the deviation of position recognition decreases because the judgment is made from the proprioceptive sensation alone. Therefore, as seen from results 3-2 and 3-3, the deviation is larger when looking at the “monkey's hand” in both Experiments 1 and 2. The validity of the hypothesis was verified by the fact that there was a significant difference between this experiment and the control experiment.

Next, we discuss the correlation between subjective data and objective data. After finding that the illusion of the loss of the thumb can be obtained from the monkey's hand position, the correlation between the intensity of the illusion and the magnitude of position recognition deviation in Experiments 1 and 2 was investigated. When examining the correlation with Experiments 1 and 2 using the average of Q1 and Q2, the subjective questions that examine the presence or absence of a four-finger sensation, there was no correlation and only a gentle positive slope. However, there was a weak correlation between Experiment 1 and each of Q1 and Q2 individually. From this, it can be concluded that at least in Experiment 1, a person who strongly felt the illusion had a larger deviation in position recognition. In addition, the fact that no correlation was found for Experiment 2 may have been affected by an error when the subjects measured the distances between fingers. More accurate analysis results could be expected if measurement methods such as laser distance meters were used instead of manual measurement.

Finally, we discuss the recognition of the thumb. Through the analysis of the lattice, the thumb hidden by the “monkey's hand” state is not simply hidden, nor is the concept of the thumb itself lost; it was considered a case in which the thumb cannot be recognized but exists somewhere. Although the body image in conventional studies is limited to the dualistic values of “existence/non-existence,” a third kind of value, “absence,” is found in our “monkey's hand” study. The image of the monkey's hand is consistent with experimental results on proprioceptive sensation. The deformation of a hand to the monkey's hand is naturally accepted by the participant, and the deviation is large under the monkey's hand condition.

Our results clarify that the illusion of the monkey's hand involves discomfort as there is ambiguity of the presence or absence of the thumb. Previous studies have assumed that discomfort as a conflict between body image and the corresponding real body is correlated with the absence of body ownership. In contrast, we assume that the discomfort is independent of body ownership. In that sense, we predict body ownership with discomfort. However, since discomfort frequently reveals the ambiguity of body ownership and disownership, which could lead to few clear experimental results, it is very difficult to clarify this illusion. After the analysis was conducted with a lattice structure, body ownership with conflict in the form of the monkey's hand illusion was clarified.

It is also reported that patients with spinal cord injury had vivid tactile sensation in their previously numb fingers, during synchronous stroking in the classical rubber hand illusion setup. It supports that subjective tactile sensation can reemerge during a simple multisensory stimulation paradigm, despite a long period of massive deafferentation (Leggenharger et al., 2013). Such recreation of a coherent mental representation of one's own body ownership might lead to compensation even for the complete absence of proprioceptive input by visual image (Fuentes et al., 2013).

Contradictory feeling of existence of absence thumb in our monkey hand's illusion is strongly relevant to the tactile sensation in numb fingers. These feeling and sensation result not from matching the vision with tactile sensation, but from the impossible matching, since thumbs in the monkey hand's illusion are not seen, and since the tactile sensation in numb fingers is lost. Even if still speculative, it suggests that bodily ownership could result not from the matching between different modalities but from compensation for the gap between the different modalities.

This is the first step toward clarifying body ownership with discomfort in the form of the deformation of body image. While clarifying a body illusion with discomfort requires lattice analysis, the data in the experiment were obtained from a subjective questionnaire. This is a limitation of our research. If the logical structure is obtained from objective data, our research can be developed further.

## 6. Conclusion

We investigated body ownership accompanied by the feeling of body deformation. Rather than a VR hand, the subject's own real hand was used for the experiment, and the body deformation led to discomfort with body ownership. Since the deformed body looks as if it were a monkey's hand, in which the thumb is far from the other four fingers, the logical status of the thumb hidden from the subject's view is ambiguous between presence and absence. Such an ambiguous feeling of body ownership is clearly seen in our lattice analysis. Most participants felt that the absence of the thumb coexisted with the other four fingers. This does not imply a simple lack of body ownership of the thumb but implies disownership of the thumb. It can be considered that the thumb does not exist in the same way as the other four fingers but exists in a specific form different from that of the other four fingers.

From the lattice analysis, it can be concluded that four fingers, namely the small, ring, middle, and index fingers, are regarded as independent fingers that can form all possible combinations. In contrast, only the thumb is separated from the other four fingers, and a pair of the thumb and other fingers cannot be imagined. This shows that the thumb is not regarded as an element of the hand but exists somewhere, and this implies the ambiguous logical status of the thumb as present and absent.

The ambiguity of the presence and absence of the thumb found in the lattice analysis is consistent with the results of Experiments 1 and 2, which show that participants felt that the palm of the hand was expanded toward the thumb and the position of the thumb was far away from the other four fingers. Thus, the illusion of the hand made it feel as if the thumb were structurally present as one element of the five fingers but functionally absent because of the small possibility of collaborating with the other four fingers. It is also shown that the stronger the illusion, the greater the deformation of the palm.

Through this research, we demonstrate the significance of discomfort accompanied by body ownership, which cannot be analyzed until the lattice structure is estimated.

## Data availability statement

The original contributions presented in the study are included in the article/supplementary material, further inquiries can be directed to the corresponding author.

## Ethics statement

The studies involving human participants were reviewed and approved by Ethics Review Committee on Research with Human Subjects, Waseda University. The patients/participants provided their written informed consent to participate in this study. Written informed consent was obtained from the individual(s) for the publication of any potentially identifiable images or data included in this article.

## Author contributions

Y-PG produced the overall design of the experiment. YR and HS conducted the experiments and analyzed the statistical tests. YR and Y-PG analyzed the experimental results in terms of the rough set lattice. YR, HS, and Y-PG wrote the manuscript. All authors contributed to the article and approved the submitted version.
